# LAPTM4b recruits the LAT1-4F2hc Leu transporter to lysosomes and promotes mTORC1 activation

**DOI:** 10.1038/ncomms8250

**Published:** 2015-05-22

**Authors:** Ruth Milkereit, Avinash Persaud, Liviu Vanoaica, Adriano Guetg, Francois Verrey, Daniela Rotin

**Affiliations:** 1 Program in Cell Biology, The Hospital for Sick Children, Biochemistry Department, University of Toronto, Toronto, Ontario M5G 0A4, Canada; 2 Center for Integrative Human Physiology (ZIHP) and NCCR Kidney, Institute of Physiology, University of Zurich, Zurich 8057, Switzerland

## Abstract

Mammalian target of rapamycin 1 (mTORC1), a master regulator of cellular growth, is activated downstream of growth factors, energy signalling and intracellular essential amino acids (EAAs) such as Leu. mTORC1 activation occurs at the lysosomal membrane, and involves V-ATPase stimulation by intra-lysosomal EAA (inside-out activation), leading to activation of the Ragulator, RagA/B-GTP and mTORC1 via Rheb-GTP. How Leu enters the lysosomes is unknown. Here we identified the lysosomal protein LAPTM4b as a binding partner for the Leu transporter, LAT1-4F2hc (*SLC7A5-SLAC3A2*). We show that LAPTM4b recruits LAT1-4F2hc to lysosomes, leading to uptake of Leu into lysosomes, and is required for mTORC1 activation via V-ATPase following EAA or Leu stimulation. These results demonstrate a functional Leu transporter at the lysosome, and help explain the inside-out lysosomal activation of mTORC1 by Leu/EAA.

## Abstract (web summary)

Essential amino acids such as leucine activate mTORC1 signalling after entering the lysosome, but the molecular basis for lysosomal amino-acid uptake is unclear. Here Milkereit *et al*. show that LAPTM4b, a lysosomal membrane protein, recruits a leucine transporter to the lysosome and promotes amino-acid influx and mTORC1 signalling.

## Graphical abstract

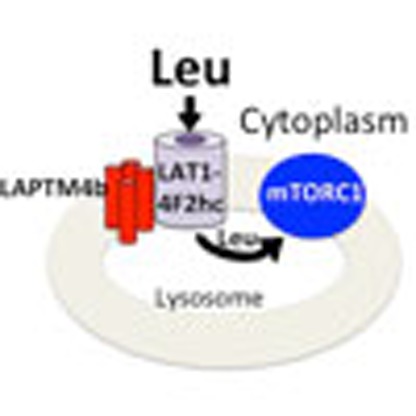

Mammalian target of rapamycin 1 (mTORC1) is a Ser/Thr kinase complex that regulates cellular and animal growth, and has been implicated in numerous diseases, including cancer^1^,^2^,^3^. mTORC1 is a key mediator of protein translation (and a suppressor of autophagy) downstream of activated growth factor receptor, energy mediators and amino-acid influx^4^,^5^. Influx of essential amino acids (EAAs), such as leucine (Leu), into cells is mediated by the LAT1-4F2hc(CD98) (*SLC7A5-SLC3A2*, or System L) transporter^6^,^7^ in exchange for Gln^8^ or other non-EAAs. This Leu entry results in the recruitment of the mTORC1 complex to the lysosomal membrane by Rag GTPases^9^,^10^. Importantly, cellular influx of Leu/EAA leads to their subsequent entry into lysosomes, where they activate the lysosomal membrane protein H^+^ATPase (V-ATPase) from inside the lysosome (inside-out activation)^11^, leading to activation of the Ragulator, RagA/B-GTP and mTORC1 via Rheb-GTP. This activation stimulates phosphorylation of S6K1 and 4E-BP, and enhancement of protein translation^12^. mTORC1 activation also leads to inhibition of AMPK and autophagy^13^. A critical unsolved question has been how Leu/EAA enters the lysosomes in the first place to promote activation of mTORC1 via V-ATPase.

The lysosomal-associated transmembrane protein 4b (LAPTM4b) is a lysosomal membrane protein that comprises four transmembrane domains. It has several splice isoforms, including a 35- and a 24-kDa variant that are identical except for a 91-residue N-terminal extension in the former^14^. The 35-kDa isoform is overexpressed in numerous tumour types and its gene is amplified in breast cancer^15^,^16^. It was proposed to promote cancer by stimulating the PI3K/Akt pathway^17^. Whether the short (24 kDa) isoform of LAPTM4b, studied here, is also involved in promoting cancer is not known. LAPTM4b is mainly localized at the lysosomal membrane, but a fraction of the protein is also found at the plasma membrane; LAPTM4b lysosomal localization is mediated, in part, by its interaction with the ubiquitin ligase Nedd4 (ref. 18).

Here we show that LAPTM4b binds to LAT1-4F2hc and recruits this Leu transporter to lysosomes, promoting Leu entry into lysosomes and activation of mTORC1 via V-ATPase.

## Results

### LAPTM4b binds the Leu transporter

**Figure 1:**
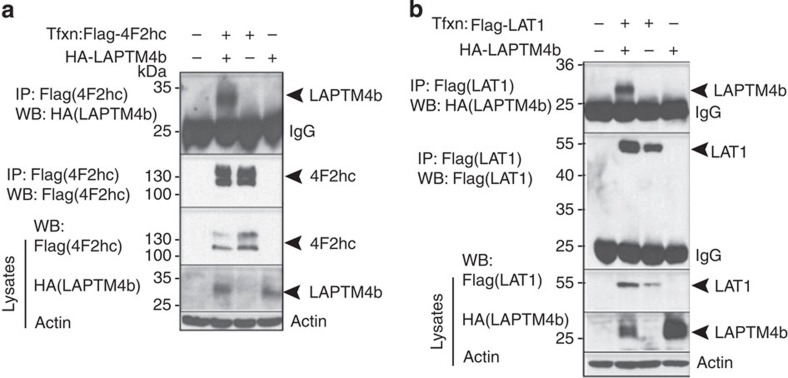
LAPTM4b binds the Leu transporter (4F2hc and LAT1). (**a**,**b**) Co-immunoprecipitation (co-IP) of LAPTM4b with 4F2hc and LAT1: HeLa cells were transfected (Tfxn) with HA-LAPTM4b and Flag-tagged (**a**) 4F2hc, or (**b**) LAT1. Following Flag IP, the presence of co-immunoprecipitated LAPTM4b was verified by immunoblotting with HA antibodies. Lower panels depict controls for the IPs, for the amounts of lysates used and for protein loading (actin).

To investigate the cellular functions of LAPTM4b, we searched for binding partners of the 24-kDa isoform using mass spectrometry, and identified 4F2hc/CD98 (*SLC3A2*) and LAT1 (*SLC7A5*) as LAPTM4b-associated proteins (Supplementary Table 1). We validated these interactions by co-immunoprecipitation (co-IP) of tagged LAPTM4b and LAT1 or 4F2hc transfected into mammalian (HeLa) cells (Fig. 1; Supplementary Fig. 1a), as well as co-IP of endogenous LAPTM4b and LAT1 (Supplementary Fig. 1b). No interaction was seen with other tested amino-acid transporters, *SLC1A5*/ASCT2 or *SLC38A2*/SNAT2, which transport Gln and other neutral amino acids (Supplementary Fig. 1c,d), suggesting that LAPTM4b does not bind nonspecifically to plasma membrane transporters. The LAPTM4b:LAT1-4F2hc interaction was not dependent on amino-acid stimulation (Supplementary Fig. 1e,f).

### LAPTM4b recruits LAT1-4F2hc to lysosomes

**Figure 2:**
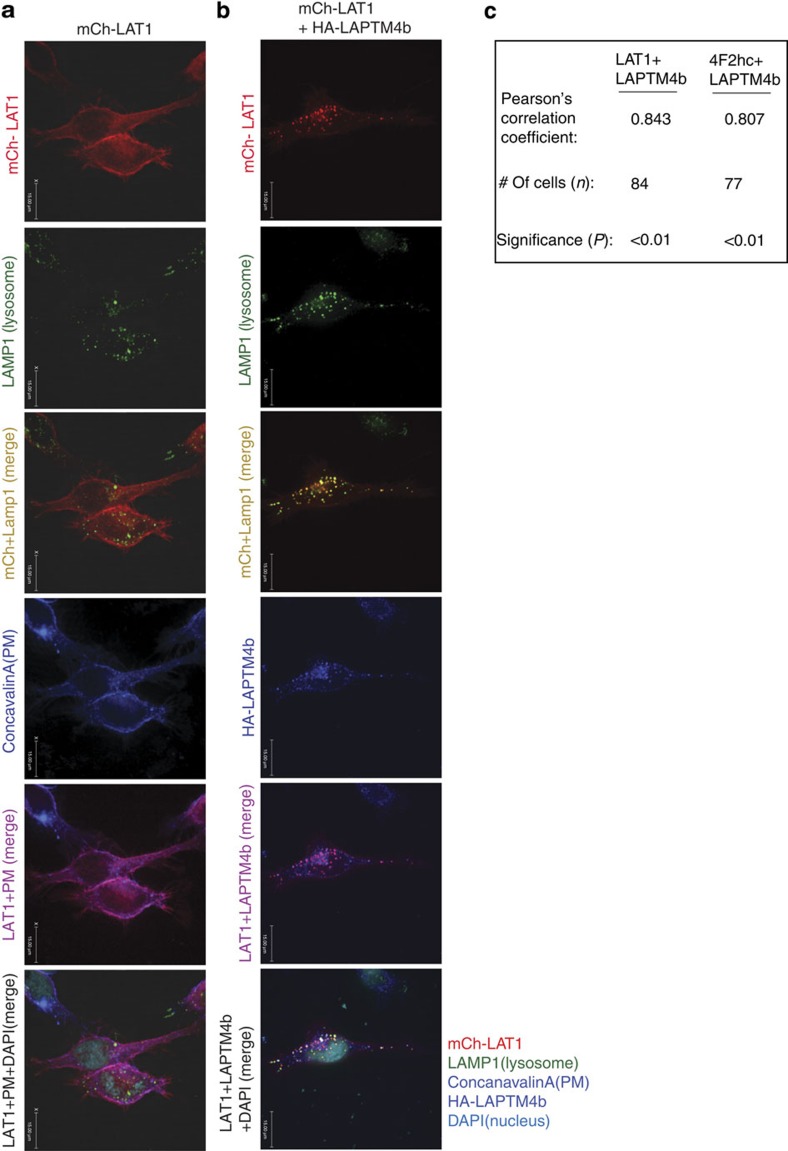
LAPTM4b recruits the Leu transporter to lysosomes. (**a**,**b**) Recruitment of LAT1 to lysosomes by LAPTM4b. HeLa cells were transfected with mCherry (mCh) LAT1 (red) alone or with HA-LAPTM4b. Cells were fixed 24 h post transfection and stained for the nucleus (4,6-diamidino-2-phenylindole (DAPI), cyan), lysosomes (LAMP1, green), plasma membrane (PM, concanavalinA, blue) and LAPTM4b (anti-HA, blue). Scale bar, 15 μm. (**c**) Quantification of co-localization of LAPTM4b with LAT1 or 4F2hc (Pearson's coefficient). The immunofluorecence imaging for 4F2hc is shown in Supplementary Fig. 2a,b. In addition to its presence in lysosomes, LAPTM4b was also detected at the plasma membrane in 54 and 49% of cells co-expressing LAPTM4b with LAT1 or 4F2hc, respectively. *P* value was calculated by the Student's *t*-test. Scale bars, 15 μm.

To study the consequence of the LAPTM4b:LAT1-4F2hc association in cells, we expressed epitope-tagged versions of these proteins in HeLa cells to trace their subcellular localization. Strikingly, while LAT1 and 4F2hc were localized to the plasma membrane, as expected, co-expression of LAPTM4b with these System L components led to the recruitment of LAT1-4F2hc to lysosomes (Fig. 2a–c; Supplementary Fig. 2a,b), with some of the Leu transporter remaining at the plasma membrane (Supplementary Fig. 2f), as expected. This lysosomal recruitment was also seen for endogenous LAT1 (Supplementary Fig. 2c–e). Accordingly, knockdown of LAPTM4b led to reduced lysosomal association of LAT1 (Supplementary Fig. 3a,b). This lysosomal recruitment of LAT1-4F2hc did not affect protein stability of LAT1 or 4F2hc, as assessed by pulse-chase analysis (Supplementary Fig. 4a–c).

### LAPTM4b promotes mTORC1 activation

**Figure 3:**
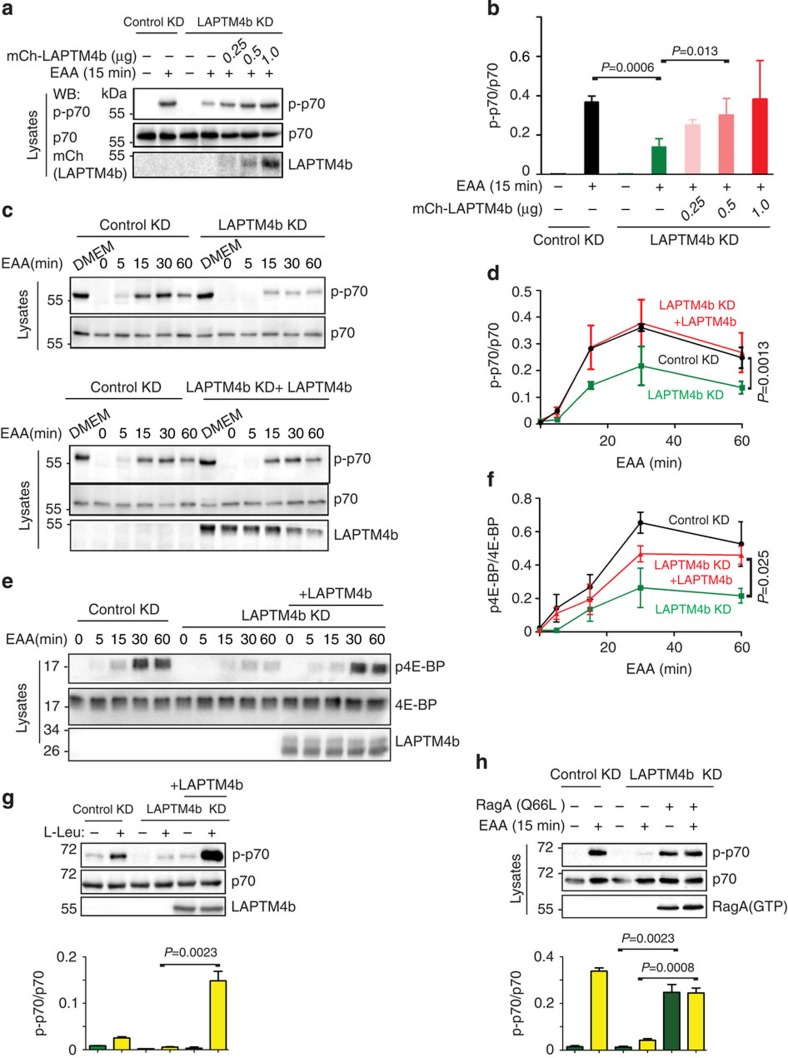
LAPTM4b stimulates mTORC1 activation. (**a**,**b**) LAPTM4b enhances mTORC1 activation: HeLa cells stably transfected with control knockdown (control KD), LAPTM4b knockdown (LAPTM4 KD, targeting its 3′untranslated region) or LAPLTM4b KD reconstituted with mCh-LAPTM4b, were serum- and nutrient-starved and tested for activation of S6K1 (pp70) following (or not) 15 min stimulation with essential amino acids (EAAs). (**a**) A representative immunoblot. (**b**) Quantification of mTORC1 activation (pp70/p70 ratio) by LAPTM4b (data are mean±s.e.m., *N*=3). (**c**–**f**) Time course of activation of mTORC1 by LAPTM4b determined by S6K1 (pp70; **c**,**d**) or 4E-BP (p4E-BP; **e**,**f**) phosphorylation in HeLa cells stably expressing control KD, LAPTM4b KD or LAPTM4b KD reconstituted with mCh-LAPTM4b (LAPTM4b KD+LAPTM4b), serum- and nutrient-starved and stimulated with EAA for the indicated times. DMEM: control (non-starved cells). (**c**,**e**) Representative immunoblots. (**d**,**f**) Quantification of p70 and 4E-BP phosphorylation (activation), respectively. (**g**) mTORC1 activation (S6K1 phosphorylation) in serum- and nutrient-starved cells by Leu (0.4 mM, 15 min) alone. The experiment was performed as in **a**. (**h**) Rescue of mTORC1 activation in cells knocked down for LAPTM4b and expressing a constitutively active RagA, RagA(Q66L). (**g**,**h**) Quantification of data is depicted underneath the immunoblots. For all panels depicting quantification: values are mean±s.e.m. (*N*=3 independent experiments). *P* values were calculated from Student's *t*-tests.

Given our observed recruitment of LAT1-4F2hc to lysosomes by LAPTM4b (Fig. 2; Supplementary Fig. 2), we tested whether expression of LAPTM4b can enhance mTORC1 activation following EAA stimulation. Thus, we generated stable HeLa cell lines with knockdown of LAPTM4b by targeting its 3′-untranslated region (Supplementary Fig. 5a). We then starved these knocked-down cells of serum and nutrients overnight, a procedure that did not affect their viability (Supplementary Fig. 5b), and stimulated them with EAA for 15–60 min. As seen in Fig. 3a–d, loss of LAPTM4b led to an inhibition of mTORC1 activation, as determined by pT389-S6K1 (pp70) phosphorylation (pp70/p70 ratio). Moreover, reintroduction of LAPTM4b into these knocked-down cells led to restoration of EAA-induced S6K1 phosphorylation, indicating that LAPTM4b was able to stimulate mTORC1 activation, an effect also observed by stimulating these cells with Leu alone (Fig. 3g). This effect was also seen with other clones of HeLa cells generated with different LAPTM4b-directed short hairpin RNAs (shRNAs) (Supplementary Fig. 5c), suggesting the results are not due to off-target effects of LAPTM4b knockdown. Furthermore, knockdown of LAPTM4b in two cancer cell lines known to overexpress LAPTM4b^15^,^16^, the breast cancer MDA-MB-231 and the hepatic carcinoma HepG2 lines, which also express high levels of endogenous LAT1 (Supplementary Fig. 6a,b), led to a marked reduction in S6K1 phosphorylation (Supplementary Fig. 6c–f). Accordingly, reconstitution of LAPTM4b in LAPTM4b-depleted MDA-MB-231 cells rescued mTORC1 activation (Supplementary Fig. 6g). These results demonstrate that the positive effect of LAPTM4b on mTORC1 activation is not confined to HeLa cells.

We also measured mTORC1 activation by analysing 4E-BP phosphorylation; 4E-BP is a downstream effector of mTORC1 and its phosphorylation releases its inhibitory association with the eukaryotic initiation factor eIF-4E, promoting the binding of eIF-4E to eIF3 and eIF4G and initiation of translation^12^. As seen in Fig. 3e,f, phosphorylation of 4E-BP was blocked following knockdown of LAPTM4b, but was restored in LAPTM4b-reconstituted cells. In accord, knockdown of LAPTM4b resulted in stimulation of autophagy (Supplementary Fig. 7). The effect of LAPTM4b on EAA-mediated mTORC1 activation occurs upstream of RagA, as evidenced by rescued activation of mTORC1 in cells depleted of LAPTM4b and reconstituted with a constitutively active RagA (RagA(Q66L), Fig. 3h). In support of earlier work that implicated Leu influx into cells in mTORC1 activation^8^, we show here that competition of LAT1-4F2hc-mediated Leu transport with D-Phe was rescued with the constitutively active RagA mutant (Supplementary Fig. 5d), although we currently do not know whether D-Phe inhibits Leu influx into cells, into lysosomes or both.

### LAPTM4b promotes Leu entry into lysosomes

**Figure 4:**
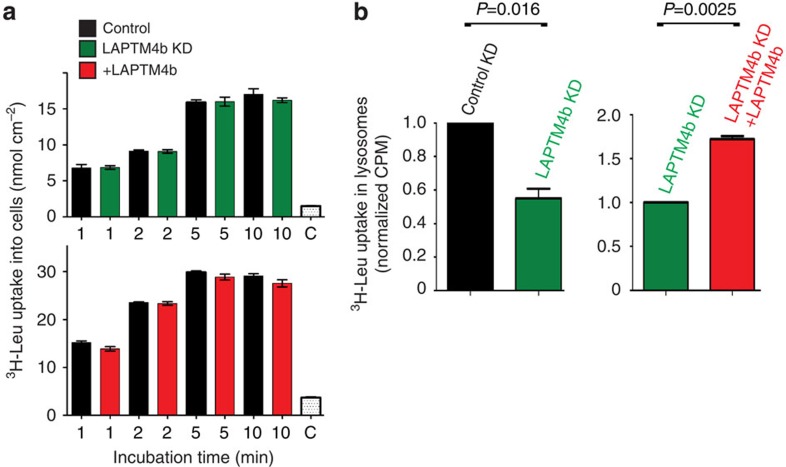
LAPTM4b promotes uptake of Leu into lysosomes. (**a**) ^3^H-Leu uptake into cells is not affected by LAPTM4b: HeLa cells stably knocked down for LAPTM4b (control and LAPTM4b KD, upper panel) or overexpressing LAPTM4b (lower panel) were incubated for 1–10 min in uptake solution containing 5 μM [^3^H]L-Leu. C (control) represents competition with 5 mM L-Leu. (**b**) ^3^H-Leu uptake into lysosomes is reduced upon knockdown of LAPTM4b and restored upon its re-expression: HeLa cells stably knocked down for control KD, LAPTM4b (LAPTM4b KD) or reconstituted with LAPTM4b (LAPTM4b KD+LAPTM4b) were transfected with Flag-LAMP1, serum- and nutrient-starved and stimulated for 10 min with EAA that contains 0.4 mM Leu plus [^3^H]-Leu. Lysosomes were isolated by anti-Flag immunoprecipitation and their radioactivity quantified by scintillation counting, as described^11^. Data are mean±s.e.m. (*N*=3 independent experiments, each performed in triplicates). *P* values were calculated from the Student's *t*-test.

To evaluate the effect of LAPTM4b on Leu transport, we tested the effect of knocking down or reconstituting LAPTM4b on ^3^H-Leu entry into cells and into lysosomes. Our results show that while knockdown or reconstitution of LAPTM4b did not affect influx of ^3^H-Leu into cells via the plasma membrane (Fig. 4a; Supplementary Fig. 8a–d), it caused a large reduction (∼50%) of [^3^H]-Leu uptake into lysosomes, which was rescued by re-expression of LAPTM4b (Fig. 4b). LAPTM4b itself does not mediate Leu transport (Supplementary Fig. 8a–d). In agreement with the ability of LAT1-4F2hc to transport Leu into lysosomes, we found that the activity of this Leu transporter is fully functional at pH 5.0, compatible with intra-lysosomal pH (Supplementary Fig. 8e).

Together, these results demonstrate that LAPTM4b recruits LAT1-4F2hc to lysosomes to promote Leu uptake into these organelles and is required for mTORC1 activation. Consistent with the above findings, LAPTM4b knockdown resulted in attenuated cell proliferation and reduced cell size, which were restored by re-expression of LAPTM4b (24 kDa) (Supplementary Fig. 9).

### LAPTM4b promotes mTORC1 activation via V-ATPase

**Figure 5:**
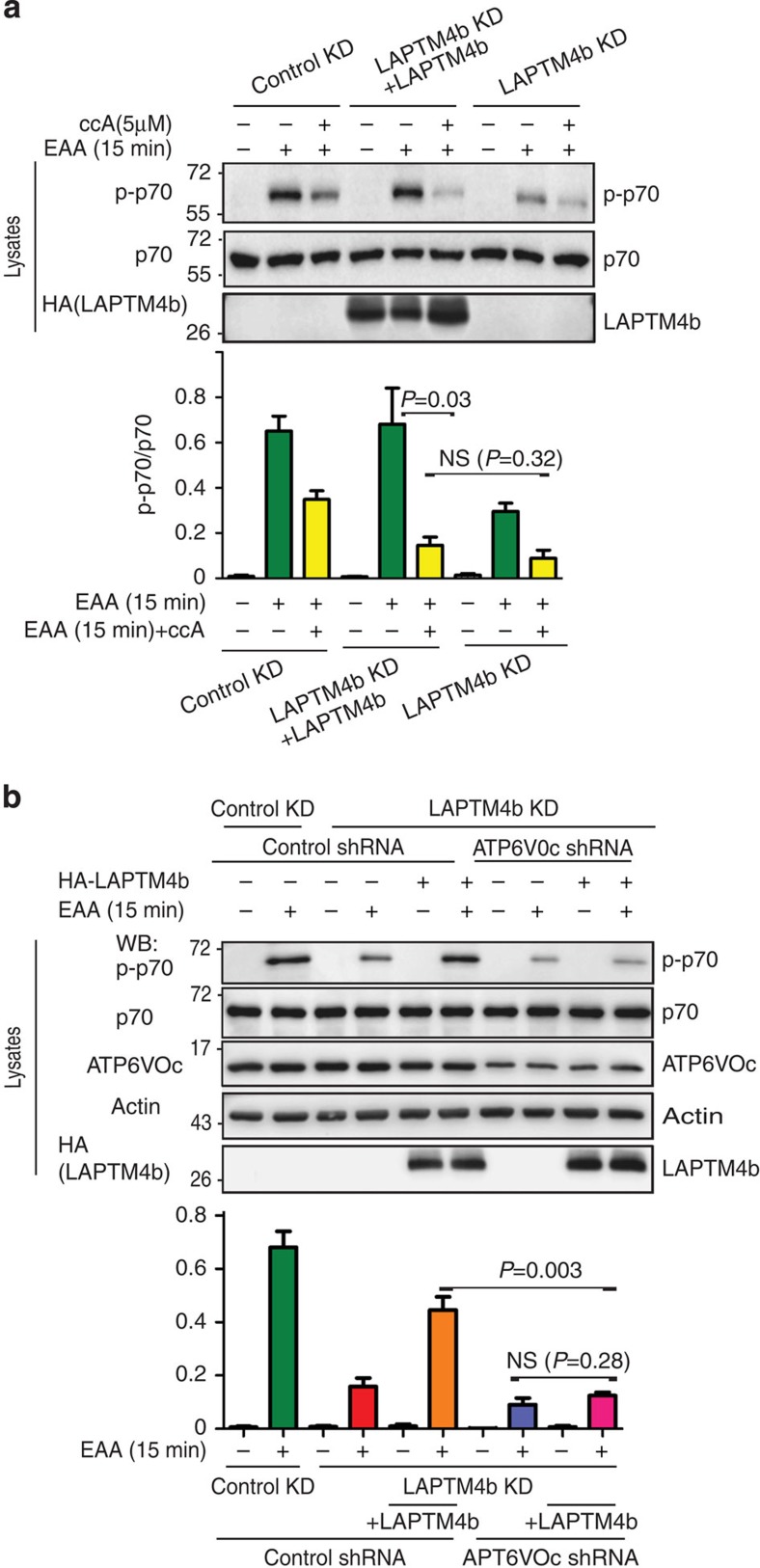
LAPTM4b requires active V-ATPase for mTORC1 activation. (**a**) HeLa cells expressing control KD, LAPTM4b KD or LAPTM4 KD reconstituted with HA-LAPTM4b (LAPTM4b KD+LAPTM4b) were serum- and nutrient-starved overnight and stimulated with EAA for 15 min in the presence/absence of the V-ATPase inhibitor Concanamycin A (ccA, 5 μM). mTORC1 activation was determined by immunoblotting for activated S6K1 (pp70), as in Fig. 3 above. The top panel depicts a representative experiment, while the bottom panel depicts the quantification of three separate experiments. (**b**) HeLa cells expressing control KD, LAPTM4b KD or LAPTM4 KD reconstituted with HA-LAPTM4b (LAPTM4b KD+LAPTM4b) were transfected with V-ATPase shRNA (ATP6VOc shRNA) to yield 60% knockdown. Cells were serum- and nutrient-starved overnight, stimulated with EAA for 15 min and analysed for S6K1 activation as described in **a**. In **b**, the top panel depicts a representative experiment, while the bottom panel depicts the quantification of three separate experiments. In **a** and **b**, values are mean±s.e.m. (*N*=3). *P* values were calculated from Student's *t*-tests. NS, not significant.

mTORC1 activation downstream of amino-acid stimulation was previously shown to be mediated via intra-lysosomal amino-acid stimulation of V-ATPase (H^+^-ATPase)^11^. Thus, we tested whether the enhancement of mTORC1 activation by LAPTM4b involves V-ATPase. We first inhibited V-ATPase with concanamycin A (ccA) and tested mTORC1 activation following EAA stimulation of starved cells either depleted (by knockdown) of LAPTM4b, or depleted and reconstituted with LAPTM4b. Figure 5a shows that inhibiting V-ATPase indeed reduced S6K1 phosphorylation (mTORC1 activation), as previously reported^11^. Interestingly, reconstitution of LAPTM4b after its knockdown was not able to properly restore mTORC1 activation in the presence of ccA (Fig. 5a). In accord, knockdown of the V-ATPase V0c subunit, which is required for function of this ATPase in mTORC1 activation^11^, prevented mTORC1 activation by LAPTM4b following EAA stimulation (Fig. 5b). The apparent small additive effect of LAPTM4b knockdown and ccA could be explained by a residual activity of the knocked-down LAPTM4b. Taken together, these data show that LAPTM4b-mediated activation of mTORC1 requires active V-ATPase, suggesting that LAPTM4b functions upstream of this H^+^ pump and RagA.

## Discussion

**Figure 6:**
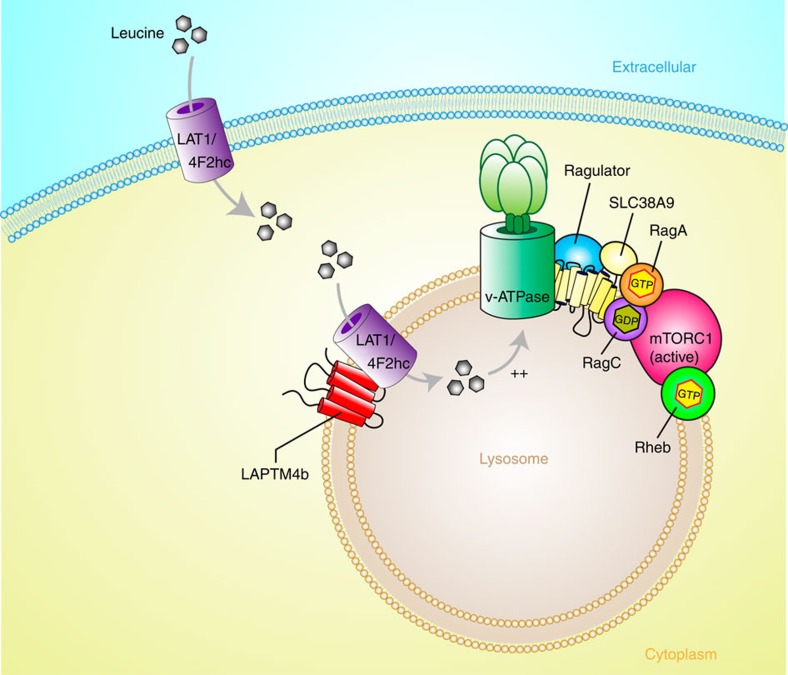
Proposed role of LAPTM4b in activation of mTORC1 via recruitment of the Leu transporter to lysosomes. The lysosomal protein LAPTM4b recruits LAT1-4F2hc (the Leu transporter) to the lysosome, promoting entry of Leu and other EAA into the lysosome, stimulating activation of V-ATPase and hence mTORC1 activation (via the Ragulator, RagA/B-GTP and Rheb-GTP).

Our work presented here demonstrates that LAPTM4b promotes Leu uptake into lysosomes and mTORC1 activation, most likely by recruiting LAT1-4F2hc to the lysosomal membrane. We show that LAPTM4b recruits the Leu transporter LAT1-4F2hc to lysosomes, enhances Leu uptake into lysosomes and stimulates mTORC1 activation via V-ATPase (Fig. 6). These findings not only identify a functional Leu transporter (System L) at the lysosomal membrane, but also help solve the puzzle of how mTORC1 is activated by amino acids in the lysosome by an inside-out mechanism (intra-lysosomal stimulation of V-ATPase) originally noted by Sabatini *et al*.^11^. Since a strong knockdown of LAPTM4b resulted in 50% reduction in lysosomal Leu uptake, it is likely that other protein(s) aside from LAPTM4b may also contribute to the recruitment of System L to lysosomes, and/or that other Leu transporters may also contribute.

The mode of interaction between LAPTM4b and LAT1-4F2hc (whether direct or indirect) is currently unknown. Our data show that it does not involve the cytoplasmic N or C termini of LAPTM4b (Supplementary Fig. 10a–f). Since removing the C terminus, which caused endoplasmic reticulum (ER) retention of both LAPTM4b and LAT1 (Supplementary Fig. 10g), did not disrupt LAPTM4b:LAT1-4F2hc binding, we suspect that this association takes place already at the ER, possibly during or soon after protein translation. We also do not know whether LAPTM4b recruits LAT1-4F2hc to lysosomes directly from the Golgi, from the plasma membrane or both.

LAT1-4F2hc is an amino-acid antiporter known to exchange large neutral amino acids, in particular essential ones such as Leu, across the plasma membrane^6^,^19^. Rate limiting for its function is generally the cytosolic concentration of substrate amino acids, because its intracellular affinity is much lower than its extracellular one. In the presence of a high intracellular concentration of non-EAAs such as Gln, EAAs may thus be accumulated in the cell via LAT1-4F2hc in exchange for the efflux of non-EAAs. On the basis of the observed LAPTM4b-mediated translocation of LAT1-4F2hc to lysosomes, we suggest that the entry of Leu into lysosomes observed here and earlier^11^ may be mediated by LAT1-4F2hc. Thus, not only a substantial cytosolic Leu concentration would be required to drive its lysosomal Leu uptake, but sufficient intra-lysosomal neutral amino acids would be necessary as exchange substrates to sustain LAT1-4F2hc exchange. Possible sources of lysosomal amino acids include hydrolysis of peptides/proteins activated by the V-ATPase-mediated H^+^ accumulation in lysosomes or uptake of large neutral (non-essential) amino acids such as Gln into lysosomes via directional/accumulative amino-acid transporters. Candidate transporters could be in particular SNAT3 (*SLC38A3*) and SNAT5 (*SLC38A5*), which co-transport Na^+^ plus non-essential neutral amino acids in exchange for H^+^ and thus also would be activated by V-ATPase-mediated H^+^ accumulation. Another candidate is the H^+^-coupled lysosomal transporter PAT1, which exports small neutral amino acids from the lysosome and was proposed to regulate mTORC1 activation^11^, although the mode of its regulation of mTORC1 is unclear^20^. The recently described SLC38A9.1, which directly binds the Ragulator and Rag proteins, is a low-affinity Arg transporter that was proposed to function instead as a transceptor to sense Arg^21^,^22^; it is not clear whether it could provide counter-transport for Leu entry into lysosomes.

While our data indicate that the translocation of LAT1-4F2hc with LAPTM4b to lysosomes plays a major role in the stimulation of mTORC1 by EAAs, they do not reveal how and where precisely amino acids such as Leu are sensed.

Finally, while the 35-kDa LAPTM4b isoform has been shown to promote cancer via binding to p85 of PI3K and PI3K/Akt activation^16^,^17^, it is possible that the 24-kDa isoform, which lacks the binding site for p85, also has a role in cancer promotion by enhancing mTORC1 activation via amino acids, a finding supported by our observed stimulation of cell growth and proliferation by this LAPTM4b isoform.

## Methods

### Cell lines and reagents including antibodies

The following antibodies (and dilutions) were used: anti-LAMP1 (H4A3, 1:1,000), anti-Giantin (ab24586, 1:2,000), anti-ATP6V0c (ab104374, 1:100) and anti-mCherry (1C51, 1:1,000) from Abcam; Anti-β-actin (#A2228, 1:10,000), anti-FLAG (#F1804, 1:10,000) and anti-Calnexin (c4731, 1:1,000) from Sigma; anti-haemagglutinin (HA) (MMS-101R, 1:1,000) from Covance, anti-HA (ab137838, 1:10,000) from Abcam, anti-pS6K (pp70, 1:1,000) (Thr389, #9234S), anti-4E-BP1 (9452, 1:1,000), anti-p4E-BP1 (T37/46, 1:1,000) (9459S) and anti-LAT1 (#5347S, 1:1,000, from Cell Signaling and KAL-KE026, 1:500, from Cosmo Bio, Japan), anti-LAPTM4b (APR62852_P050, 1:1,000, from Aviva Bio; EC-1 (1:1,000) from Abgent (a gift from Dr Rou Li Zhou; and 18895-1-AP, 1:1,000, from Proteintech), anti-S6K (p70) (SC-8418, 1:1,000) and anti-CD98/4F2hc (C-20) (sc-7095, 1:500) from Santa Cruz; anti-LC3 (NB600–1384, 1:2,000) from Novus Biologicals; anti-FLAG M2 affinity agarose (A2220) and anti-HA agarose (#26181) were from Sigma and Pierce, respectively. Secondary antibodies conjugated to horseradish peroxidase (1:10,000) were purchased from Molecular Probes. Reagents for imaging: normal goat serum (Jackson ImmunoResearch Laboratories Inc.), Alexa-Fluor 647 conjugated ConcanavalinA (Invitrogen), Alexa-Fluor 488/647 Goat anti-mouse or anti-rabbit 2° antibody (Invitrogen) all used at 1:1,000 and 4,6-diamidino-2-phenylindole (Molecular Probes, 1:5,000). Radioactive Leucine (Leu) uptake was monitored with [^3^H]-Leu (PerkinElmer). Cell lines used originated from ATCC, unless indicated otherwise. HeLa, MDA-MB-231 and HepG2 cells were maintained in full media (DMEM supplemented with 10% fetal bovine serum, 100 U ml^−1^ penicillin and 100 μg ml^−1^ streptomycin), and BT549 cells were maintained in RPMI supplemented as for the other cells. Cells were transfected using Polyjet reagent (Signagen). For stimulation experiments, cells were serum- and nutrient-starved overnight in RPMI 1640 medium without amino acids, sodium phosphate powder (#R8999-04A, US Biological) and stimulated with RPMI supplemented with 1 × MEM (minimal essential medium (or EAA), Life Technologies, with amino-acid composition provided in: http://www.lifetechnologies.com/ca/en/home/technical-resources/media-formulation.164.html); uncropped blot images are shown in Supplementary Fig. 11.

### cDNA constructs

Human ORFeome complementary DNA (cDNA) entry clones (pDONR223) for LAPTM4b (AAH31021.1), LAT1/SLC7A5 (BC039692), 4F2hc/SLC3A2 (BC003000) and LAMP1 (BC021288) were obtained from SIDNET/SPARC (SickKids). Each construct was N-terminally tagged with either Flag or mCherry (mCh) using the Gateway cloning system (Invitrogen). LAPTM4b truncation mutants: ΔC-LAPTM4b (amino acids 1–181), ΔN-LAPTM4b (amino acids 21–226) and ΔNΔC-LAPTM4b (amino acids 21–181) were sub-cloned from LAPTM4b wild-type cDNA into pcDNA3.1-nHA using the Gateway system. Constitutively active CFP-tagged Rag A (Q66L) was obtained from Dr John Brumell (Hospital for Sick Children).

### Generation of stable LAPTM4b knockdown cell lines

HeLa cells were transfected with either one of three LAPTM4b-specific shRNAs: V2LHS_175452 (targets LAPTM4b open-reading frame (ORF), V3LHS_340114 (targets ORF), V3LHS_405603 (targets 3′ untranslated region) or nonspecific shRNA all in the pGIPZ vector from Open Biosystems. V3LHS_405603 (shRNA 603) was used in all experiments, unless otherwise indicated. The cells were maintained in growth media supplemented with 1 μg ml^−1^ puromycin until colonies formed. For generating stable LAPTM4b knockdown in MDA-MB-231, HepG2 and BT549 cells lines: MDA-MB-231, HepG2 and BT549 cells were transfected with either shRNA to knockdown LAPTM4b (V3LHS_405603) or pGIPZ-Ctrl (control). Transfected cells were selected in Puromycin (0.25 μg ml^−1^ for MDA-MB-231 and 1 μg ml^−1^ for HepG2 and BT549) in DMEM+fetal bovine serum and antibiotics. Green fluorescent protein-positive colonies were selected and expanded as individual clones. LAPTM4b knockdown efficiency was monitored by quantitative PCR using the LAPTM4b unique primers from Integrated DNA Technologies (Supplementary Table 2).

### Affinity-tag immunoprecipitation and mass spectrometry

The protocol designed for affinity-tag IP of HA-LAPTM4b-WT and sample preparation for tandem mass spectrometry (MS) was modified from a previously established protocol^23^. Briefly, HeLa cells were transiently transfected with HA-LAPTM4b-WT for 48 h. Cells were lysed on ice in lysis buffer (50 mM Hepes, pH 7.5, 150 mN NaCl, 1% Triton X-100, 10% glycerol, 1.5 mM MgCl_2_, 1.0 mM EGTA, supplemented with 10 μg ml^−1^ leupeptin, 10 μg ml^−1^ aprotinin, 10 μg ml^−1^ pepstatin and 1 mM phenylmethanesulfonylfluoride) and HA-LAPTM4b was immunoprecipitated with anti-HA agarose beads at 4 °C for 3 h. Beads were washed three times with IP wash buffer (20 mM Hepes, pH 7.5, 150 mM NaCl, 10% glycerol and 0.1% Triton X-100) and twice with HPLC-grade H_2_O. Bound proteins were eluted with 0.1% trifluoroacetic acid and peptides were generated by trypsin digestion (Biolab, TPCK, #P8101S). Peptide fragments were desalted using Pierce C-18 Spin Columns (Thermo Scientific, #89870, Lot#NJ176772). The eluted peptide mix was analysed by shotgun liquid chromatography tandem mass spectrometry (MS/MS). All MS/MS samples were analysed using Sequest (Thermo Fisher Scientific, San Jose, CA, USA; version 1.3.0.339) and X! Tandem (The GPM, thegpm.org; version CYCLONE (2010.12.01.1)). Scaffold (version Scaffold_3.6.4, Proteome Software Inc., Portland, OR) was used to validate MS/MS-based peptide and protein identifications.

### Immunofluorescent confocal microscopy

HeLa cells were cultured on poly-D-lysine-coated coverslips in six-well plates and transiently transfected with the indicated cDNA. At 24 h post transfection, wells were washed three times with cold 1 ml PBS and incubated for 5 min with Alexa-Fluor-647-conjugated ConcanavalinA (1:1,000) on ice to visualize the plasma membrane. The cells were fixed with 4% paraformaldehyde, permeabilized with 0.1% Triton X-100 and incubated with 1:100 normal goat serum in 3% skim milk (30 min). Slides were stained for 1 h with either rabbit anti-HA (1:1,000), mouse anti-human LAMP1 (1:1,000), rabbit anti-human Giantin (1:2,000) or rabbit anti-human Calnexin (1:1,000) in 3% skim milk. After three PBS washes, cells were incubated with goat anti-mouse or anti-rabbit Alexa 488/647 Fluor-conjugated 2° antibody and briefly stained with 4,6-diamidino-2-phenylindole. Coverslips were mounted with Dako Cytomation. Images were acquired using a Quorum WAveFX-X1 spinning disc confocal system at × 60 magnification with an Olympus S-Apo × 60/1.35 oil objective (Quorum Technologies Inc., Guelph, Canada). Co-localization was assessed by Volocity 6.0.1 (PerkinElmer) and expressed as Pearson's correlation coefficients.

### Co-immunoprecipitation assays

HeLa cells were co-transfected with the specified cDNA constructs and lysed in lysis buffer. Co-IP of FLAG-LAT1, FLAG-4F2hc, mCherry-SNAT2 or mCherry-ASCT2 with HA-LAPTM4b was determined by IP of either FLAG-LAT1 or FLAG-4F2hc from 1 mg of cleared cell lysate with either anti-Flag M2 affinity beads and immunoblotting with anti-HA antibody, or IP of HA-LAPTM4b with anti-HA antibody coupled to Protein G Sepharose (Bioshop) and immunoblotting with anti-mCherry antibody. Endogenous Co-IP of LAT1 and LAPTM4b was performed by IP of LAT1 from 1.5 mg of cleared HeLa cell lysate and immunoblotting with anti-LAPTM4b antibodies. Co-IP of HA-LAPTM4b ΔN, ΔC and ΔNΔC mutants with FLAG-4F2hc was determined by IP of LAPTM4b as above, and immunoblotting with anti-FLAG antibody.

### Amino-acid stimulation and mTORC1 activation

Cells were serum-starved overnight in RPMI 1,640 medium without amino acids and were restimulated with RPMI supplemented with EAA (or MEM) or 0.4 mM L-leucine (LEU222.25, Bioshop) for the indicated times. For knockdown of ATP6V0c experiment, HeLa cells were transfected with either control or ATP6V0c-715 siRNA (Abcam) and HA-LAPTM4b 48 h prior to serum starvation. For the ccA experiments, cells were transfected with HA-LAPTM4b where indicated and then treated with 5 μM ccA (Sigma) for 1 h prior to stimulation. For Rag A (Q66L) rescue experiments, HeLa cells were transfected with CFP-Rag A (Q66L) for 48 h, starved for 1.5 h and stimulated with EAA with or without 20 mM D-phenylalanine (P1751, Sigma) for the indicated times. Cells were lysed in lysis buffer and mTORC1 activation was monitored using anti-pT389-S6K1 and anti-p4E-BP1 antibodies. All blots were imaged using the Odyssey Imaging system and quantified using Image Studio version 3.1.4 (LI-COR).

### Leu uptake by lysosomes

HeLa cells stably expressing control, LAPTM4b knockdown (KD), or LAPTM4b KD reconstituted with LAPTM4b, were grown in four 10-cm dishes and transfected with FLAG-Lamp1. At 24 h post transfection, cells were starved overnight in RPMI and then stimulated with RPMI supplemented with MEM (EAA, which includes 0.4 mM Leu) and 4 μCi [^3^H]-Leu per 5 ml per dish. Samples were incubated for 10 min, at which point the amount of Leu taken up by the lysosomes was measured, as described^11^. Briefly, for each cell type (control KD or LAPTM4b KD), cells from all four plates were pooled to generate a post-nuclear supernatant fraction by lysing cells through a 23-G needle attached to a 1-ml syringe in fractionation buffer (50 mM KCL, 90 mM K-gluconate, 1 mM EGTA, 5 mM MgCl_2_, 50 mM sucrose, 20 mM HEPES, pH7.4, supplemented with protease inhibitors (leupeptin, pepstatin and aprotinin, 10 μg ml^−1^). Samples were spun for 10 min at 425*g* (4 °C). The post-nuclear supernatant was divided into 3 aliquots and spun at 20,000*g* for 15 min at 4 °C. The resulting subcellular fraction (pellet) that contains the FLAG-bound lysosomes was resuspended and topped up to 1 ml of fractionation buffer. Thirty μl anti-FLAG M2-affinity agarose was added to each sample and incubated at 4 °C for 2 h. Samples were spun for 1 min at 425*g* and washed three times with cold fractionation buffer. The affinity beads with bound lysosomes were transferred to scintillation vials using cut pipet tips and [^3^H]-Leu radioactivity was measured with a scintillation counter (Hidex 300 SL, Southern Scientific) using the MiKrowin 2,000 software (Mikrotek Laborsysteme GmbH). 100% counts in lysosomes ranged from ∼40 to 130 counts per min (similar to the values described by Zoncu *et al*.^11^). The experiment was repeated three times, each in triplicates. Successful enrichment for the lysosomal fraction was monitored by SDS–polyacrylamide gel electrophoresis of lysates at various stages of the isolation.

For oocyte experiments, [^3^H]-Leu uptake was carried out after injection of cRNA encoding a fusion protein of LAT1-4F2hc into oocytes as detailed in ref. 24.

### Cell proliferation and cell size and viability assays

HeLa cells (control KD, LAPTM4b KD or mCherry-LAPTM4b reconstituted into LAPTM4b KD cells) were seeded at 4,500 cells per well in a 96-well plate in duplicates, and cell proliferation was measured for the indicated times using the Alamar Blue assay (Invitrogen) as per the manufacturer's instructions. Absorbance at 600 nm was measured using the Spectra Max 190 plate reader (Molecular Devices). All absorbance readings were normalized to day 0. For the cell size assay, HeLa cells (control KD, LAPTM4b KD or mCherry-LAPTM4b reconstituted into the LAPTM4b-depleted cells) were seeded at 30,000 cells per well in a 24-well plate in duplicates and cell size was measured using the Cellomics VTI (Thermo Fisher) and a modified Target Activation algorithm. Individual cells were identified and defined by green fluorescent protein fluorescence, and the image pixels were used to determine cell size for individual cells. For mCherry-LAPTM4b-reconstituted cells, expression of RFP was used to gate selected cells such that only cells with a predetermined level of LAPTM4b-RFP fluorescence were included in measurements for cell size. At least 10,000 cells were measured per treatment, and the mean for the number of cells analysed was calculated for each treatment. For the viability assay, HeLa cells were starved overnight and cell viability was determined using the Trypan Blue stain (Invitrogen, T10282) and quantitated using the Countess Automated Cell Counter (Life Technologies).

### Cycloheximide pulse-chase experiments

HeLa cells (control KD, LAPTM4b KD or mCherry-LAPTM4b reconstituted into LAPTM4b KD cells) were treated with 50 μM cycloheximide for the indicated times. Cells were lysed and the stability of endogenous LAT1 and 4F2hc was monitored by immunoblotting. The relative abundance of each protein was normalized to actin. Images were quantified in Image Studio version 3.1.4 (LI-COR).

### Autophagy experiments

HeLa cells stably knocked down for LAPTM4b (or control) were starved and treated with 50 μM chloroquine for the indicated times, lysed as described above and autophagic rate (flux) was monitored using LC3 antibodies. All blots were imaged using the Odyssey Imaging system and quantified using Image Studio version 3.1.4 (LI-COR). For autophagy immunofluorescent experiments, LAPTM4b stable knockdown and control cells were starved for 2 h in the presence of 50 μM chloroquine, fixed with 4% paraformaldehyde and permeabilized for 15 min with cold 95% methanol. Cells were blocked for 1 h with 1% BSA (in PBS) and incubated overnight in LC3 antibody (1:200) at 4 °C before being processed for imaging using Volocity, as above.

### Data analysis

For all quantitative experiments (unless otherwise indicated), the Student's *t*-test (unpaired, two tailed) was used.

## Additional information

**How to cite this article:** Milkereit, R. *et al*. LAPTM4b recruits the LAT1-4F2hc Leu transporter to lysosomes and promotes mTORC1 activation. *Nat. Commun.* 6:7250 doi: 10.1038/ncomms8250 (2015).

## Supplementary Material

Supplementary Information Supplementary Figures 1-11, Supplementary Tables 1-2
