# Supplementary material for: LAPTM4b recruits the LAT1-4F2hc Leu transporter to lysosomes and promotes mTORC1 activation
Source: Nat Commun. 2015 May 22;6:7250. doi: 10.1038/ncomms8250 (PMC4455107; doi:10.1038/ncomms8250)
Supplement: Supplementary Information — Supplementary Figures 1-11, Supplementary Tables 1-2 [file ncomms8250-s1.pdf]

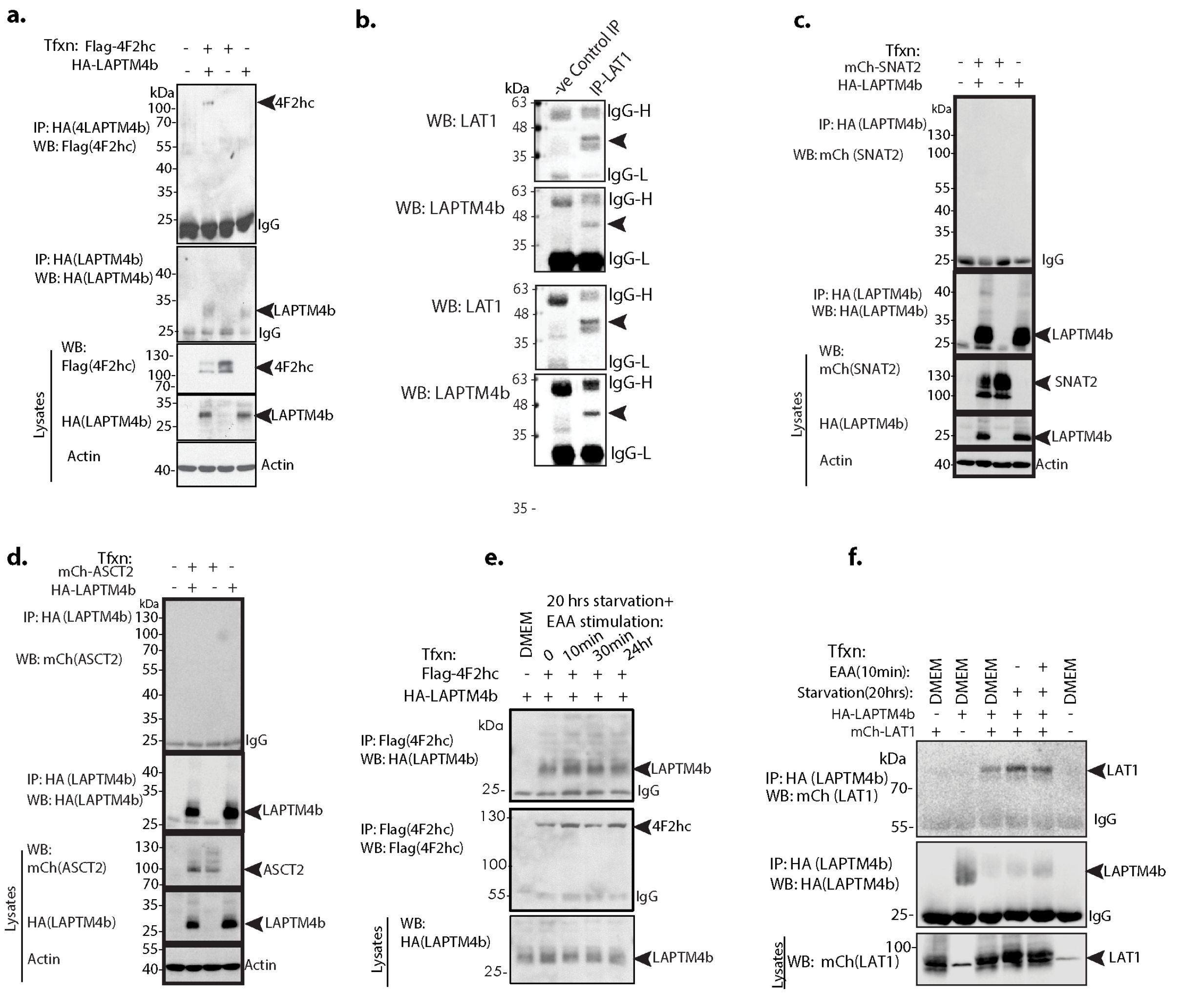

Supplementary Figure 1: **LAPTM4b binds 4F2hc (but not ASCT2 or SNAT2), and its binding to LAT1 or 4F2hc is independent of essential amino acids availability.** (**a**) Co-immunoprecipitation (co-IP) of 4F2hc with LAPTM4b: HeLa cells were transfected (Tfxn) with HA-LAPTM4b and Flag-4F2hc. Following HA IP, the presence of co-immunoprecipitated 4F2hc was verified by immunoblotting with Flag antibodies. Lower panels depict controls for the IPs, for the amounts of lysates used and for protein loading (Actin). (**b**) Co-IP of endogenous LAPTM4b with endogenous LAT1. We obtained the same results using 2 different LAT1 antibodies and 3 different LAPTM4b antibodies (directed to different epitopes of LAPTM4b), two of which shown here. (**c,d**) Lack of co-IP of the Gln transporters SNAT2 (**c**) and ASCT2 (**d**). The experiments were performed as in (**a**), except cells were transfected with HA-LAPTM4b and mCherry (mCh) tagged SNAT2 or ASCT2. (**e,f**) Binding of LAPTM4b to 4F2hc (**e**) or LAT1 (**f**) is not dependent on availability of amino acids: Cells transfected with HA-LAPTM4b and Flag-4F2hc or mCh-LAT1 were serum- and nutrient-starved for 20 hrs and stimulated (or not) with EAA for the indicated times. Co-IP experiments were done as in (**a**) above. DMEM: cells grown in full media (controls).

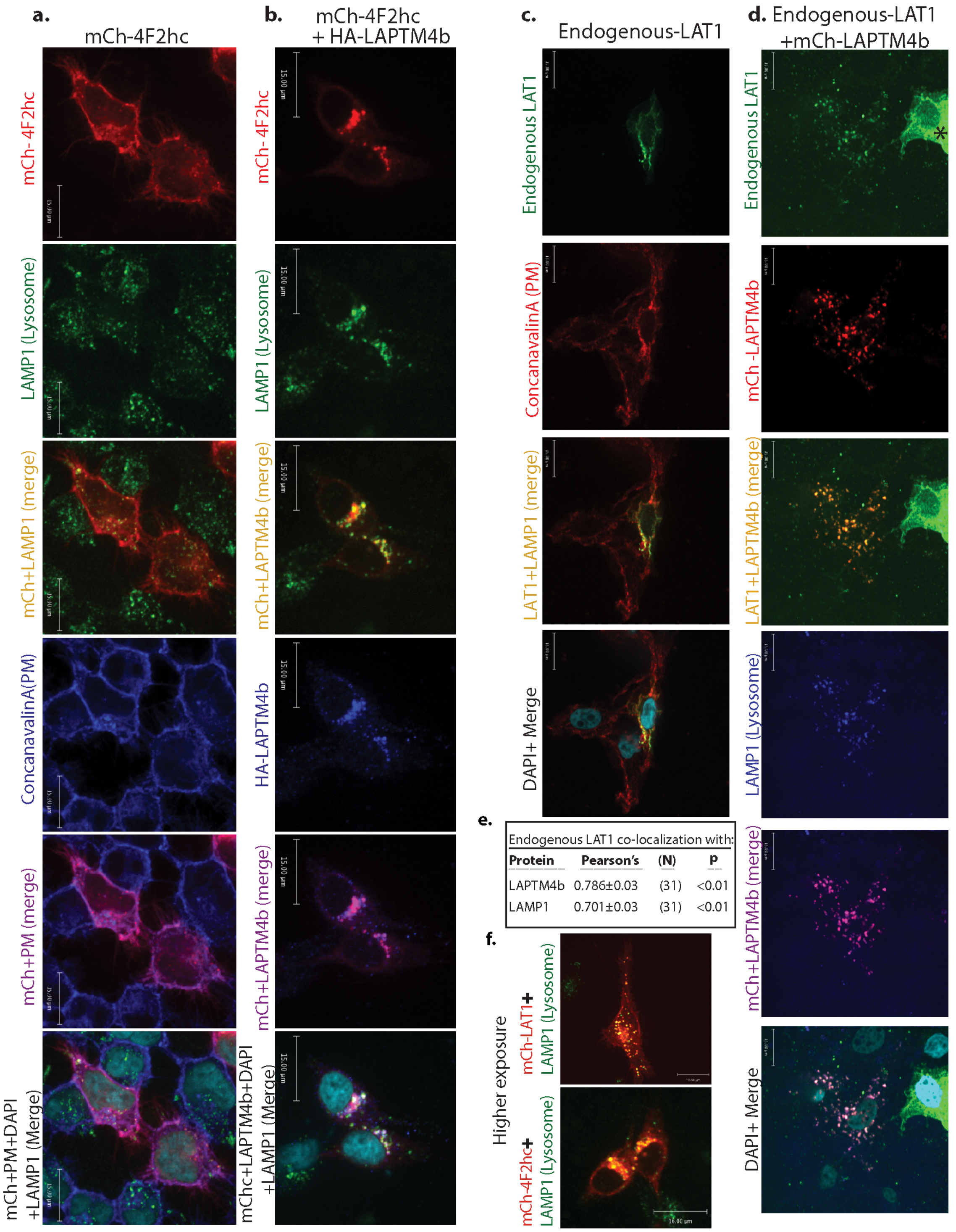

e.

Endogenous LAT1 co-localization with:

Protein	Pearson's	(N)	p
LAPTM4b	0.786±0.03	(31)	<0.01
LAMP1	0.701±0.03	(31)	<0.01

Supplementary Figure 2: **LAPTM4b recruits 4F2hc and endogenous LAT1 to lysosomes**. (**a,b**) HeLa cells transfected with mCherry (mCh) 4F2hc alone (**a**) or with HA-LAPTM4b (**b**), were fixed 24hr post transfection and stained for the nucleus (DAPI), lysosomes (LAMP1), plasma membrane (PM, concanavalinA) or LAPTM4b (HA). mCh-4F2hc is in red. Size bar: 15 μm. (**c,d**) LAPTM4b recruits endogenous LAT1 to lysosomes: HeLa cells were un-transfected (**c**) or transfected with HA-LAPTM4b (**d**). Cells were fixed 24hr post transfection and stained as indicated. Size bar: 15 μm. (**e**) Quantification of co-localization of endogenous LAT1 with LAPTM4b and lysosomes (Pearson's coefficient) is indicated in the box. The asterisk in the upper right panel in (**d**) depict an un-transfected cell. (**f**) LAPTM4b recruits a substantial amount of LAT1 and 4F2hc to lysosomes, but some LAT1-4F2hc remains at the plasma membrane.

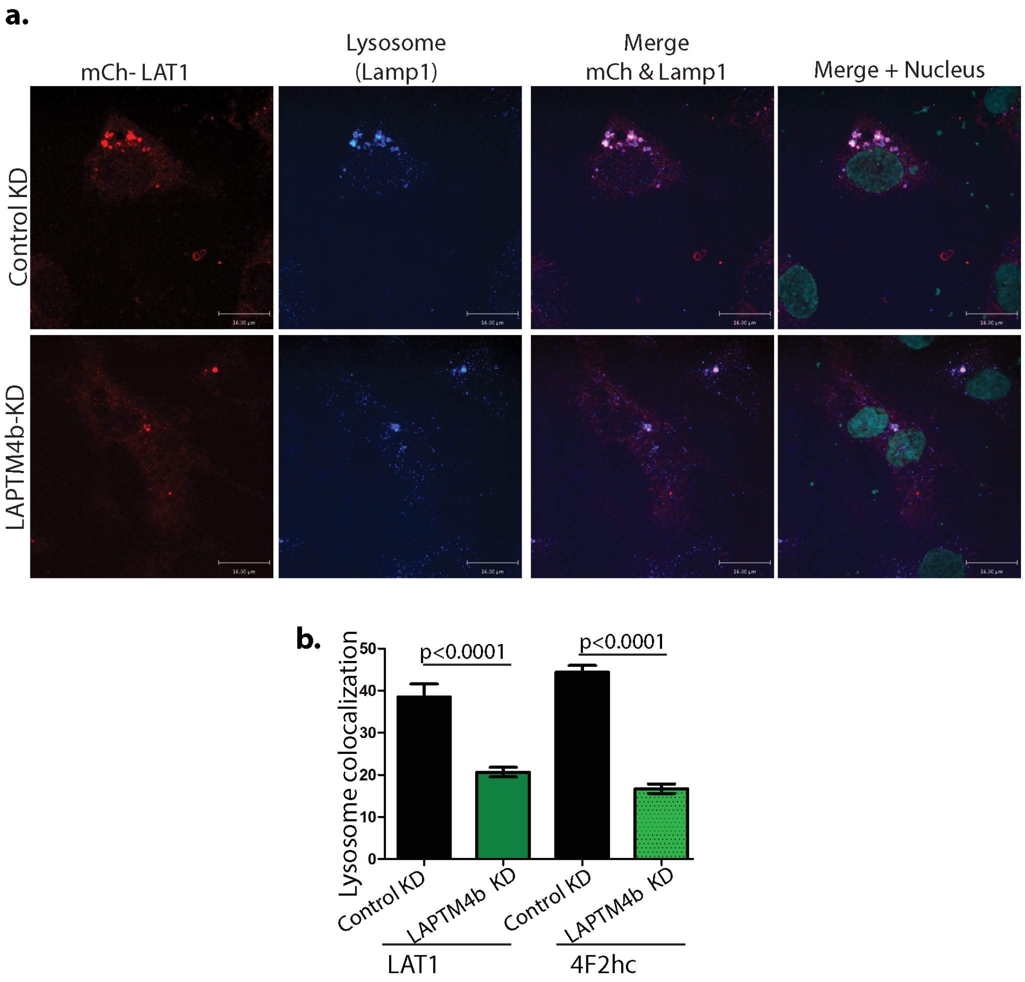

Supplementary Figure 3: **Knockdown of LAPTM4b reduces lysosomal recruitment of LAT1-4F2hc**. **(a-b):** Reduced lysosomal localization of mCh-LAT1 or mCh-4F2hc in HepG2 cells depleted of LAPTM4b by shRNA. N=41-43 cells per treatment. P<0.001, unpaired student's *t*-test. Example images for LAT1 are shown in panel (**a**). Siza bar: 16 µm.

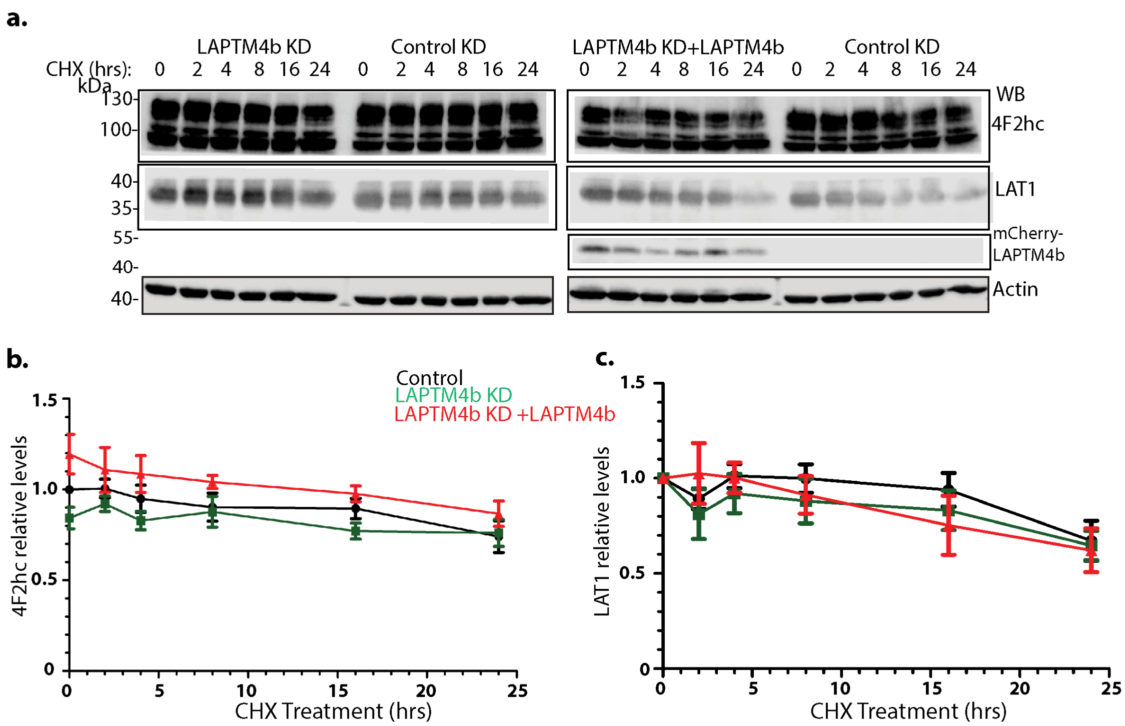

Supplementary Figure 4: **Knockdown of LAPTM4b does not affect stability of LAT1 and 4F2hc**. (**a-c**) HeLa cells stably knocked-down for LAPTM4b (LAPTM4b KD), or LAPTM4b KD reconstituted with LAPTM4b (LAPTM4b KD+LAPTM4b), or control KD, were grown in full media (DMEM) and levels of LAT1 and 4F2hc following cycloheximide (CHX) treatment determined at the indicated times by immunoblotting (**a**) and quantified (**b,c**) relative to actin loading control levels. Data are mean±SEM for 3 independent experiments

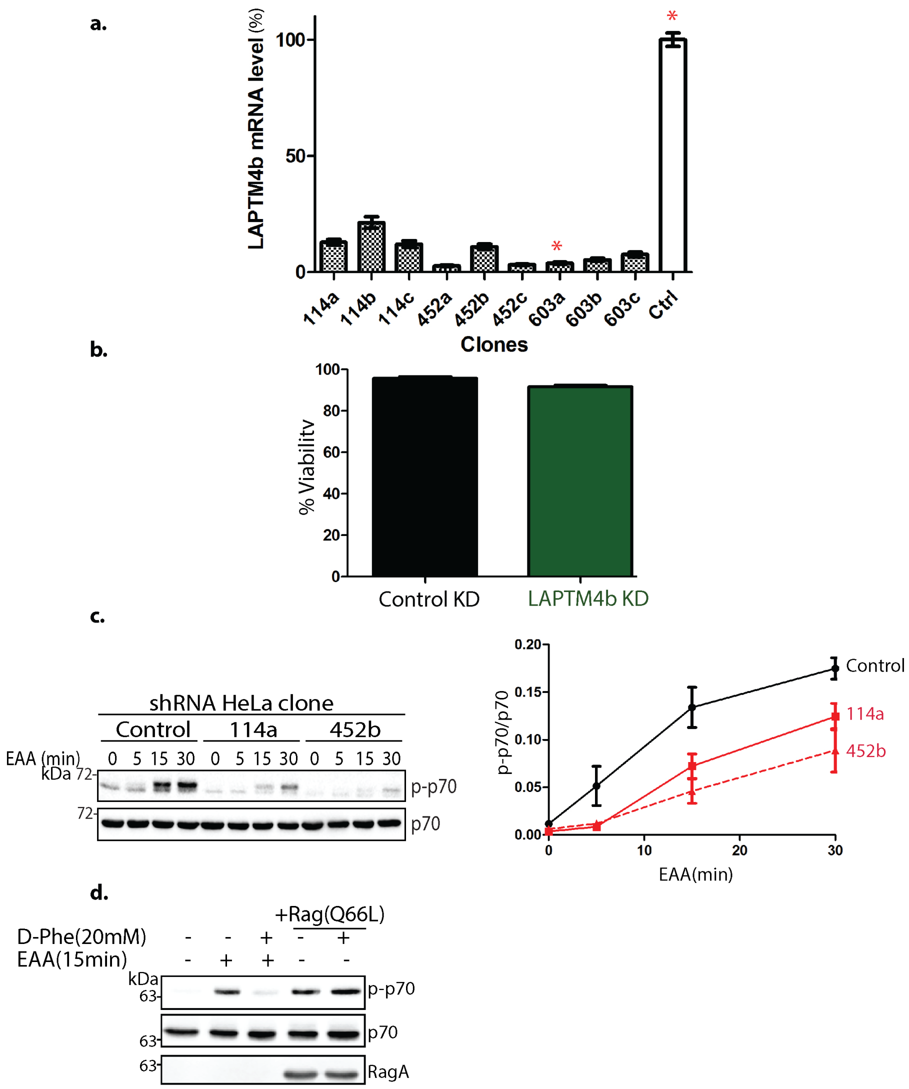

Supplementary Figure 5: **Stable knockdown (KD) of LAPTM4b does not affect cell viability after overnight starvation, and LAPTM4b stimulates mTORC1 activation by EAA following LAPTM4b knockdown with different shRNAs.** (**a**) Generation of stable LAPTM4b knockdown cells lines: HeLa cells were transfected with one of three independent pGIPZ vectors encoding shRNA to knockdown LAPTM4b (V2LHS_175**452** (targeting ORF), V3LHS_340**114** (targeting ORF), V3LHS_405**603** (targeting 3'UTR)) or pGIPZ-Ctrl (Control). The extent of LAPTM4b KD was quantified by qPCR. LAPTM4b mRNA levels are expressed relative to LAPTM4b mRNA levels in the pGIPZ-Ctrl cell line. Clone 603a and the control KD (red asterisks) clones were used in all studies, unless otherwise indicated. (**b**) Overnight serum and nutrient starvation of LAPTM4b KD or control KD HeLa cells did not affect their cell viability. Data are mean±SEM for 3 independent experiments. (**c**) mTORC1 activation (p-p70) in the indicated knockdown clones was tested in cells starved overnight and then stimulated for the indicated times with EAA, as described in Figure 2. Left panel: a representative immunoblot. Right panel: Summary of 3 separate experiments (mean±SEM). (**d**) Rescue of mTORC1 activation by RagA(Q66L) following inhibition of LAT1 with 20 mM D-Phe.

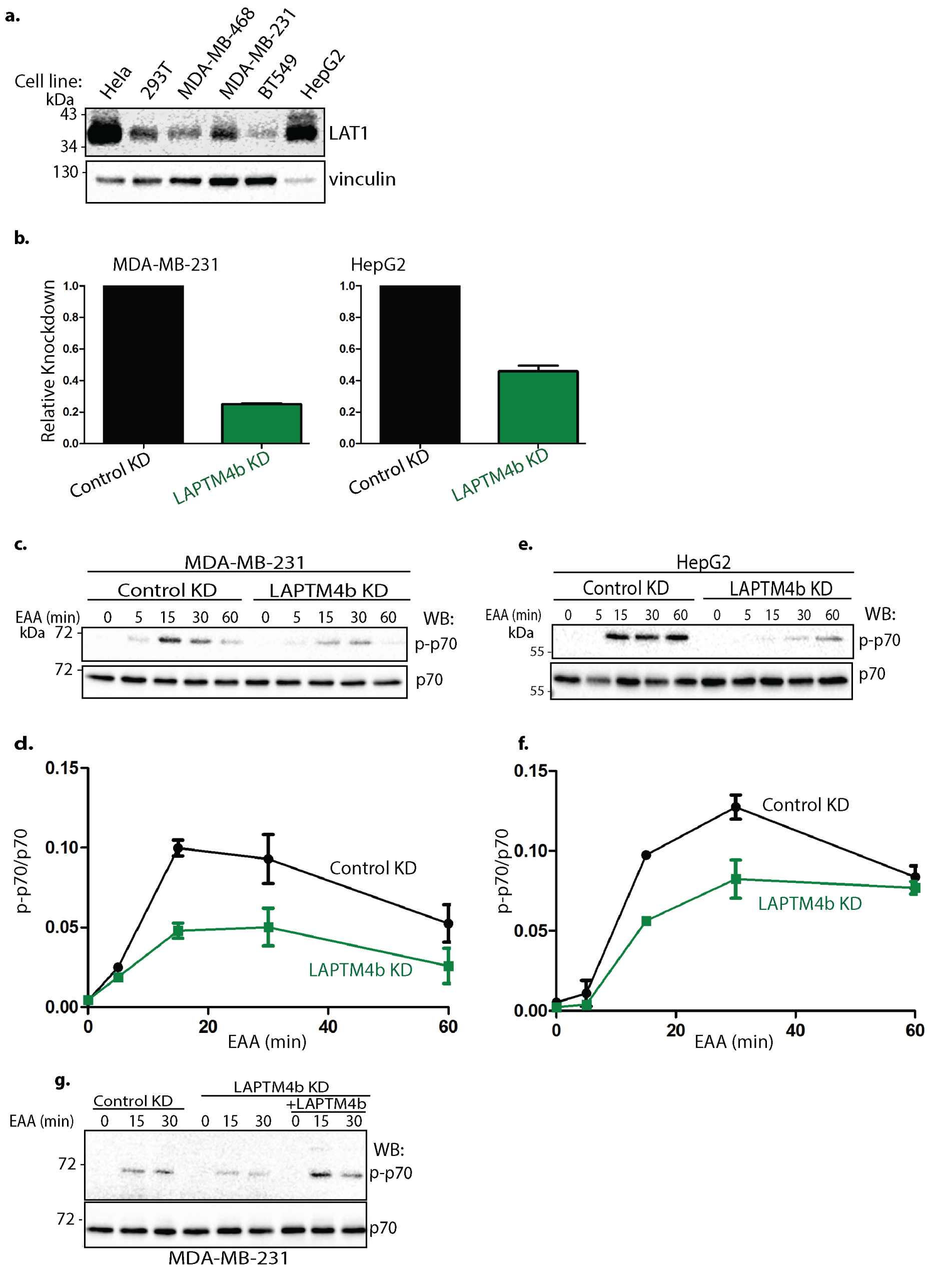

Supplementary Figure 6: **Leu-mediated activation of mTORC1 via LAPTM4b in cancer cell lines**. (**a**) Upper panel: Expression of endogenous LAT1 in the indicated cell lines. Lower panel: Vinculin loading control. (**b**) Stable knockdown of LAPTM4b in MDA-MB-231 and HepG2 cancer cells lines, analyzed by qPCR. LAPTM4b mRNA levels are expressed relative to the LAPTM4b mRNA levels of the pGIPZ-Control cell line (mean±SEM, N=3). (**c**) Immunoblot showing mTORC1 activation (p-p70) by essential amino acids (EAA) in MDA-MB-231 cells stably knocked-down (KD) for LAPTM4b, and (**d**) quantification of experiments shown in **c** for MDA-MB-231 cells (mean±SEM, N=3 independent experiments). (**e**). Immunoblot showing mTORC1 activation (p-p70) by EAA in HepG2 cells stably knocked-down (KD) for LAPTM4b, and (**f**) quantification of the experiments in HepG2 cells (mean±SEM, N=3 independent experiments). (**g**) Reconstitution of LAPTM4b into MDA-MB-231 cells depleted of LAPTM4b leads to re-activation of mTORC1 (S6K1 phosphorylation).

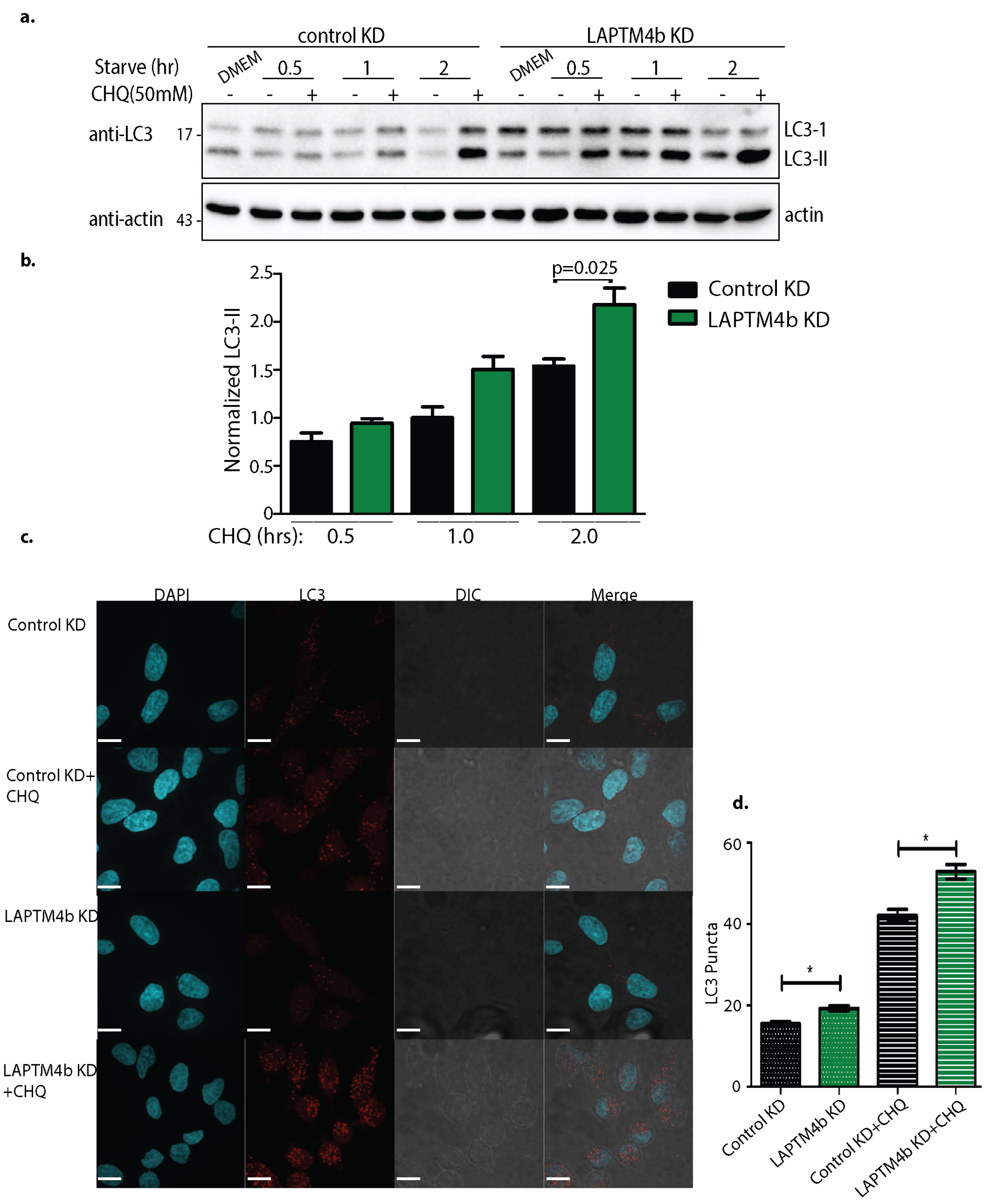

Supplementary Figure 7: LAPTM4b knockdown increases autophagy. (**a**) HeLa cells (control and LAPTM4b KD) were serum and nutrient starved in the absence or presence of 50 µM chloroquine (CHQ) for the indicated times and immunoblotted for LC3 (upper panel) and actin (lower panel). LC3-II stabilization upon CHQ treatment represents autophagy rate (flux). (**b**) Quantitation of (**a**). Data are mean±SEM from N=3 independent experiments. (**c**) HeLa cells (control and LAPTM4b KD) were serum and nutrient starved and treated with 50 µM CHQ for 2 h before fixing and staining for LC3 and DAPI (nucleus). Size bar: 15 µm. (**d**) Quantification of the data in panel C (scored blindly). Values are mean±SEM, N=100 cells per condition. P<0.0001 (Student's *t*-test).

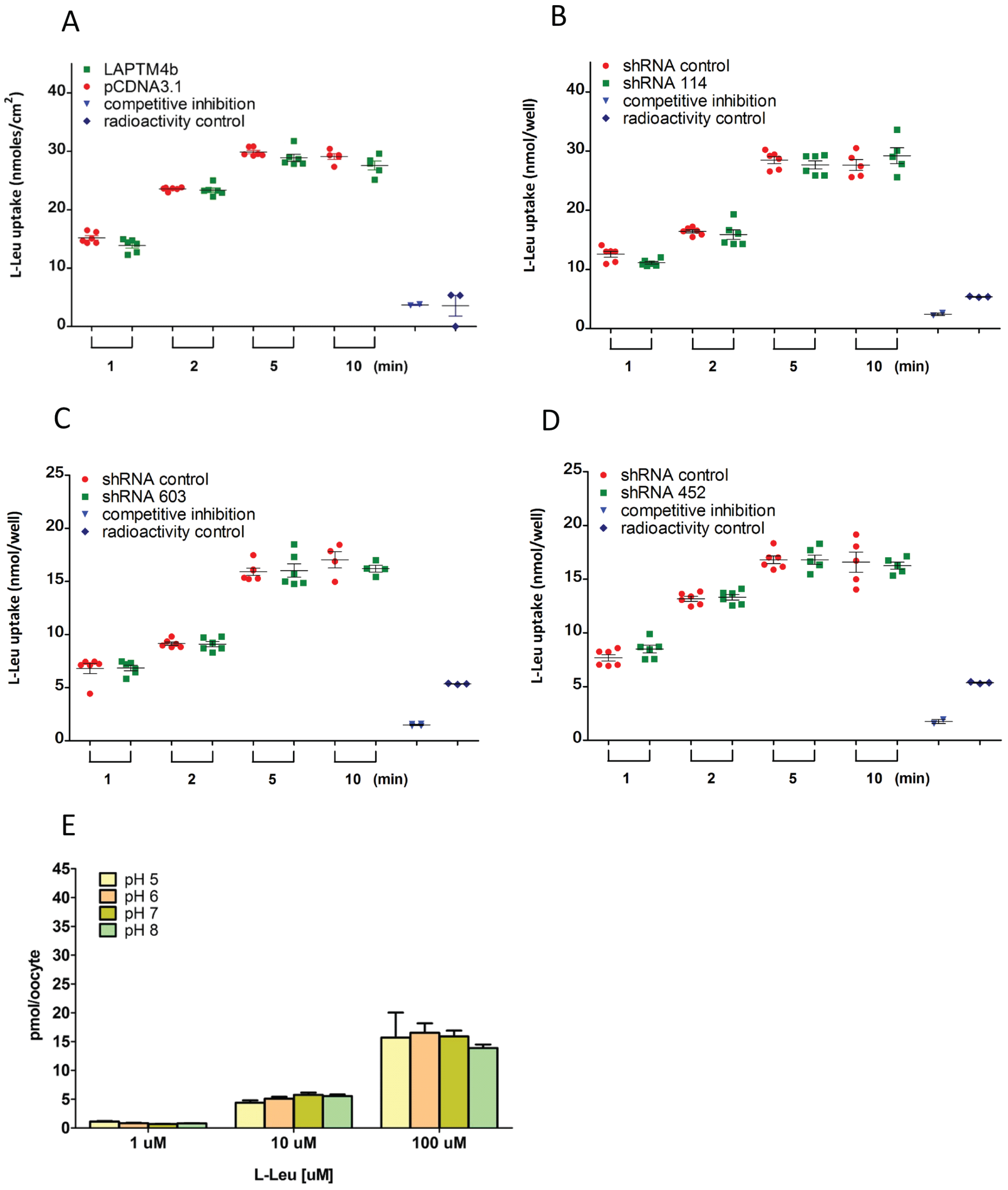

Supplementary Figure 8: **[^{3}H]-Leu uptake into HeLa cells or *Xenopus* oocytes is not affected by LAPTM4b or pH.** [^{3}H]-Leu uptake was measured at the indicated time points in HeLa cells over-expressing empty vector pCDNA3.1 or LAPTM4b (**a**) and in cells expressing control shRNA or the indicated shRNAs against LAPTM4b (**b-d**) (mean ± SEM; n = 4-6; representative of 3 independent experiments). (**e**) [^{3}H]-Leu uptake in oocytes (injected with cRNA for LAT1-4F2hc) at pH 5-8: Data are mean ± SEM of 27-30 oocytes from 3 independent experiments (mean uptake of non-injected oocytes subtracted). SEM was calculated with error propagation (SEM of test and non-injected oocytes).

a.

b.

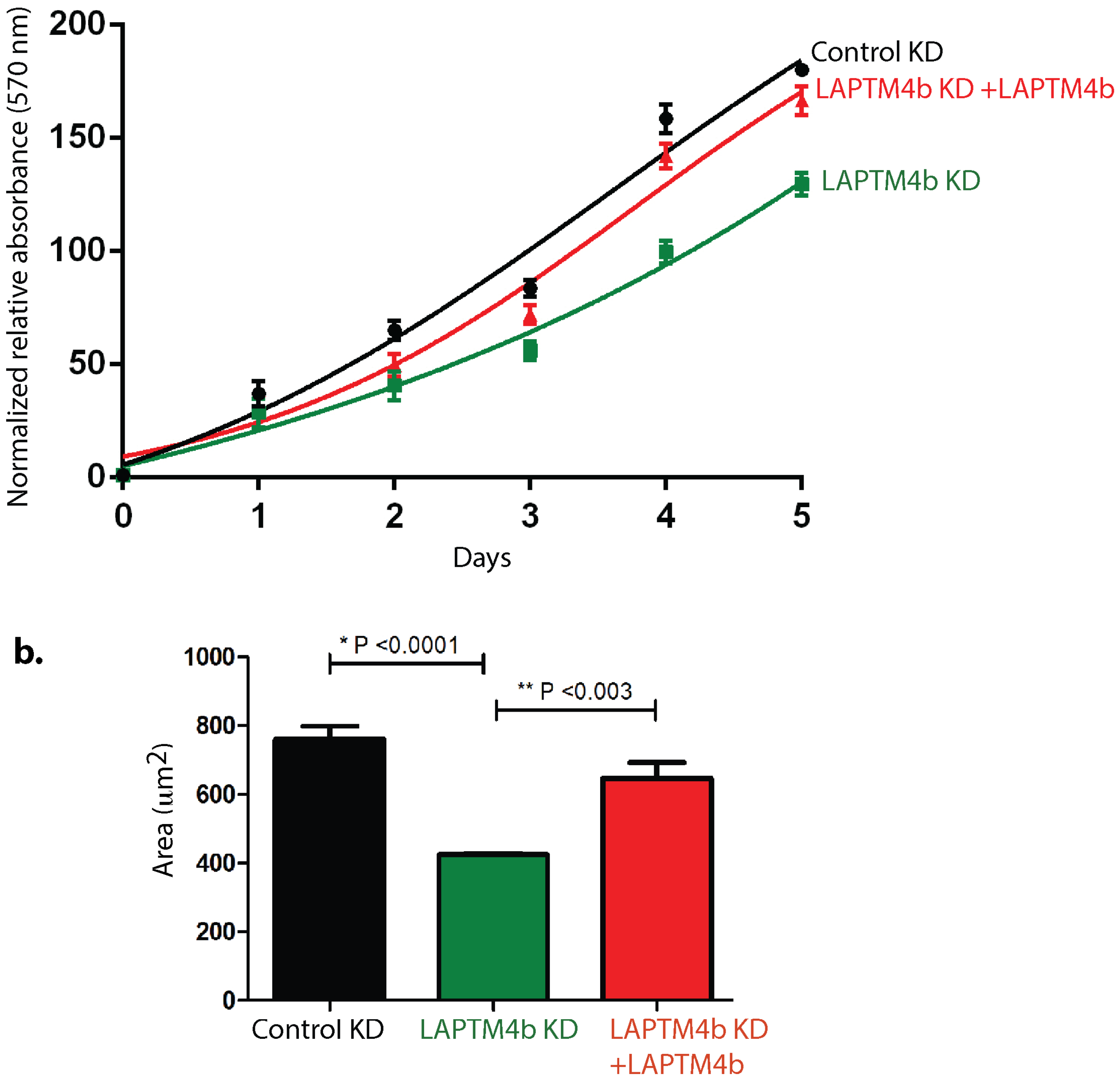

Supplementary Figure 9: LAPTM4b is a positive regulator of cell proliferation and growth. **(a)** HeLa cell proliferation in Control KD, LAPTM4b KD and LAPTM4b KD +LAPTM4b reconstituted HeLa cells was determined over a 5 day period, measured by the colorimetric Alamar Blue assay.
Data are mean±SEM for 3 independent experiments. (**b**) Reduced (HeLa) cell size (area) by LAPTM4b knockdown and reconstitution of cell size by re-expression of LAPTM4b. (mean±SEM, N>10,000 cells per treatment, student's *t*-test).

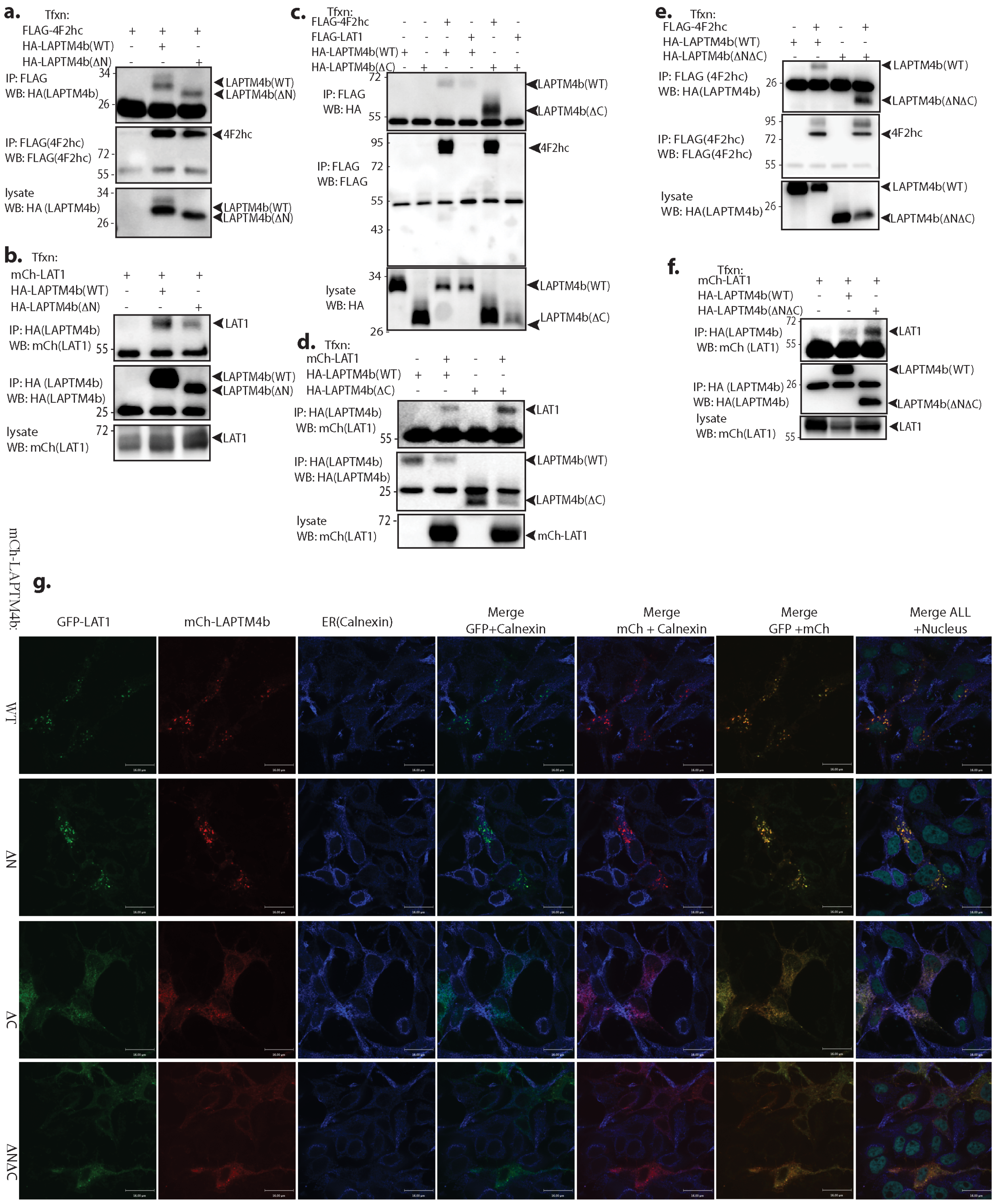

a.
Tfxn:
FLAG-4F2hc
HA-LAPTM4b(WT)
HA-LAPTM4b(ΔN)
IP: FLAG
WB: HA(LAPTM4b)
LAPTM4b(WT)
LAPTM4b(ΔN)
IP: FLAG(4F2hc)
WB: FLAG(4F2hc)
4F2hc
lysate
WB: HA (LAPTM4b)
b.
Tfxn:
mCh-LAT1
HA-LAPTM4b(WT)
HA-LAPTM4b(ΔN)
IP: HA(LAPTM4b)
WB: mCh(LAT1)
LAT1
IP: HA (LAPTM4b)
WB: HA(LAPTM4b)
lysate
WB: mCh(LAT1)
c.
Tfxn:
FLAG-4F2hc
FLAG-LAT1
HA-LAPTM4b(WT)
HA-LAPTM4b(ΔC)
IP: FLAG
WB: HA
LAPTM4b(WT)
LAPTM4b(ΔC)
IP: FLAG
WB: FLAG
4F2hc
lysate
WB: HA
d.
Tfxn:
mCh-LAT1
HA-LAPTM4b(WT)
HA-LAPTM4b(ΔC)
IP: HA(LAPTM4b)
WB: mCh(LAT1)
IP: HA(LAPTM4b)
WB: HA(LAPTM4b)
lysate
WB: mCh(LAT1)
mCh-LAT1
e.
Tfxn:
FLAG-4F2hc
HA-LAPTM4b(WT)
HA-LAPTM4b(ΔNΔC)
IP: FLAG (4F2hc)
WB: HA(LAPTM4b)
LAPTM4b(ΔNΔC)
IP: FLAG(4F2hc)
WB: FLAG(4F2hc)
lysate
WB: HA(LAPTM4b)
f.
Tfxn:
mCh-LAT1
HA-LAPTM4b(WT)
HA-LAPTM4b(ΔNΔC)
IP: HA(LAPTM4b)
WB: mCh (LAT1)
IP: HA (LAPTM4b)
WB: HA(LAPTM4b)
lysate
WB: mCh(LAT1)
g.
mCh-LAPTM4b:
GFP-LAT1
mCh-LAPTM4b
ER(Calnexin)
Merge GFP+Calnexin
Merge mCh + Calnexin
Merge GFP +mCh
Merge ALL +Nucleus
WT
ΔN
ΔC
ΔNΔC

Supplementary Figure 10: **The N and C termini of LAPTM4b do not mediate binding to 4F2hc or LAT1, and LAPTM4b C terminus targets LAT1 to lysosomes**. (**a-f**) HeLa cells were co-transfected (Tfxn) with Flag-4F2hc or mCherry (mCh)-LAT1 and LAPTM4b or its truncation mutants (LAPTM4b-ΔN, panels (**a,b**); LAPTM4b-ΔC, panels (**c,d**); or LAPTM4b-ΔNΔC, panels (**e,f**). Co-immunoprecipation (Co-IP) and immunoblotting (Western blotting, WB) were carried out as indicated. (**g**) HeLa cells expressing GFP-LAT1 and mCherry (mCh) tagged LAPTM4b-WT, LAPTM4b-ΔN, LAPTM4b-ΔC, or LAPTM4b-ΔNΔC, were immunostained for calnexin to detect ER retention. Size bar: 16 μm.

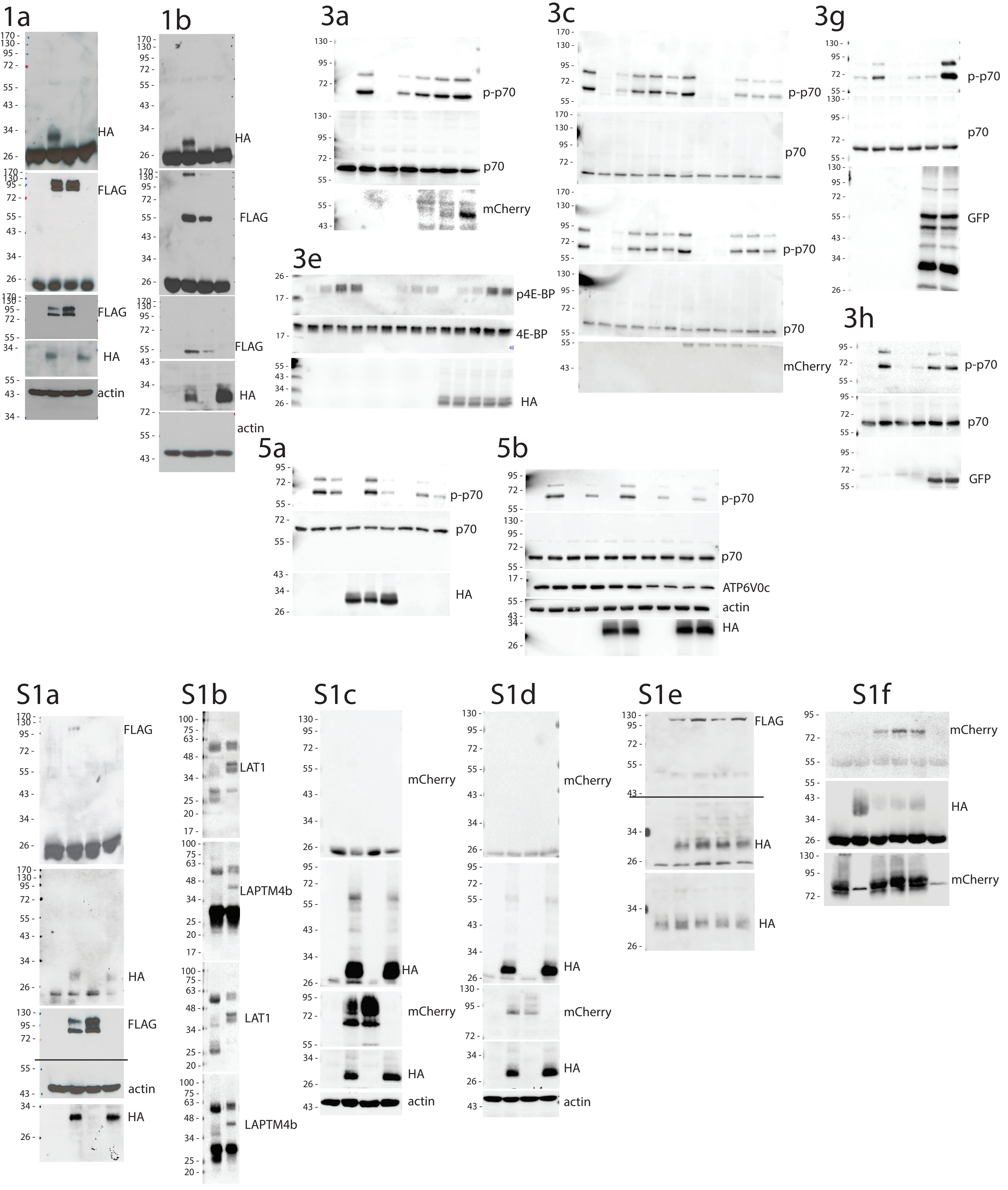
1a
HA
FLAG
FLAG
HA
actin
1b
HA
FLAG
FLAG
HA
actin
3a
p-p70
p70
mCherry
3c
p-p70
p70
p-p70
p70
mCherry
3g
p-p70
p70
GFP
3e
p4E-BP
4E-BP
HA
3h
p-p70
p70
GFP
5a
p-p70
p70
HA
5b
p-p70
p70
ATP6V0c
actin
HA
S1a
FLAG
HA
FLAG
actin
HA
S1b
LAT1
LAPTM4b
LAT1
LAPTM4b
S1c
mCherry
HA
mCherry
HA
actin
S1d
mCherry
HA
mCherry
HA
actin
S1e
FLAG
HA
HA
S1f
mCherry
HA
mCherry

S4a

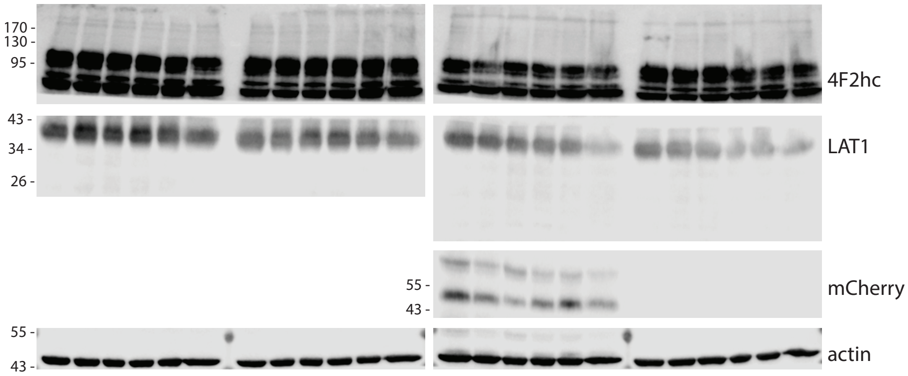

S5c

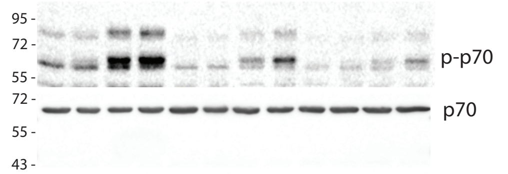

S5d

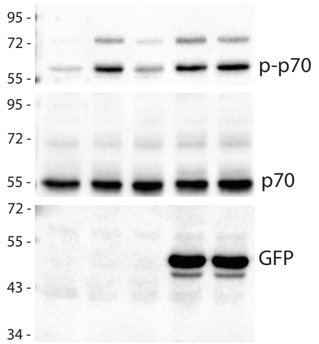

S6a

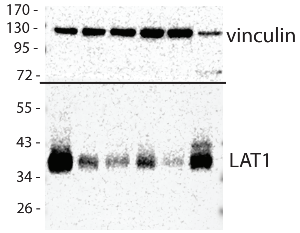

S6c

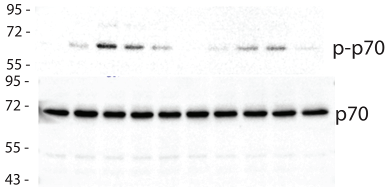

S6g

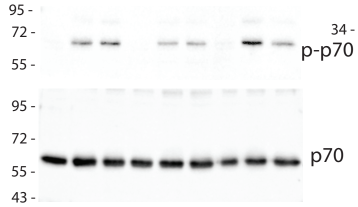

S7a

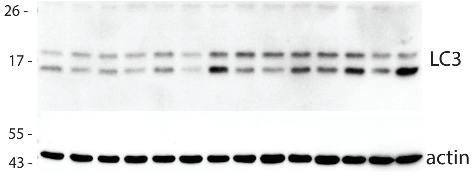

S6e

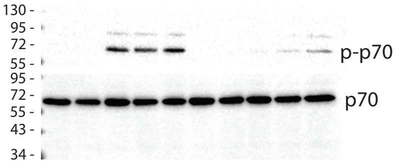

S10e

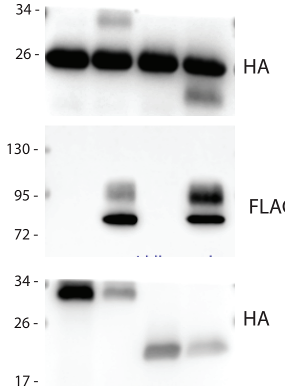

S10a

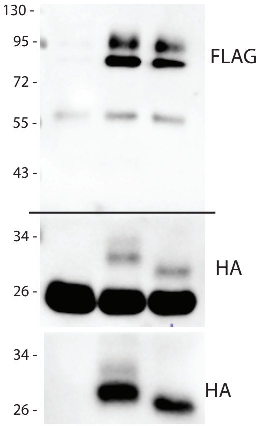

S10b

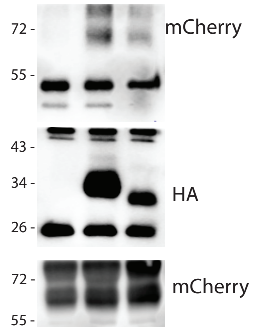

S10c

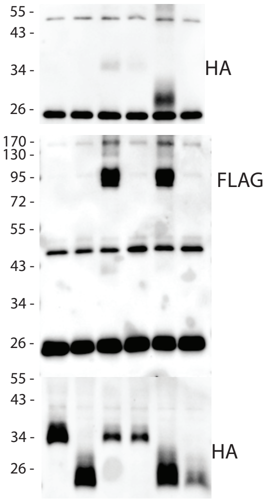

S10d

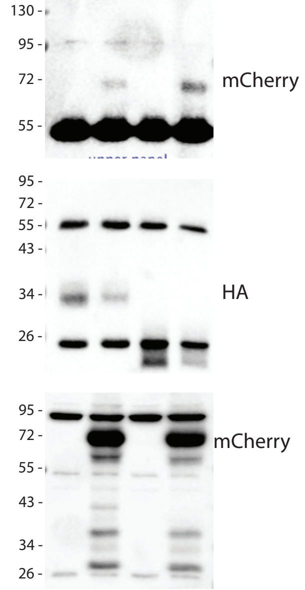

S10f

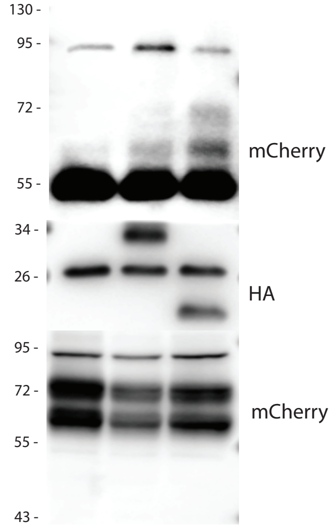

Supplementary Figure 11. Original blot images These images correspond to blot images in the main or supplementary figures as indicated.

Supplementary Table 1: Top Hits for novel LAPMT4b binding partners from two LC-MS/MS dataset

GENE	Protein Name	TOTAL ASSIGNED SPECTRA		Accession Number
		RUN1	RUN2	
HSPA8*	Heat shock cognate 71 kDa protein	39	36	sp|P11142|HSP7C_HUMAN
TUBB*	Tubulin beta chain	31	29	F8VW92_HUMAN
SLC3A2	4F2 cell-surface antigen heavy chain	14	12	F5GZS6_HUMAN
UBB*	Ubiquitin	22	18	B4DV12_HUMAN
KRT5*	Keratin, type II cytoskeletal 5	6	14	K2C5_HUMAN
LAPTM4B	Isoform 2 of Lysosomal-associated transmembrane protein 4B	29	29	sp|Q86VI4-2|LAP4B_HUMAN
TECR	Trans-2,3-enoyl-CoA reductase	9	11	sp|Q9NZ01|TECR_HUMAN
SLC25A2	ADP/ATP translocase 2	11	12	ADT2_HUMAN
SLC25A3	Isoform B of Phosphate carrier protein, mitochondrial	8	7	sp|Q00325-2|MPCP_HUMAN
AUP1	Isoform Short of Ancient ubiquitous protein 1	6	5	sp|Q9Y679-2|AUP1_HUMAN
HSPA1B*	Heat shock 70 kDa protein 1A/1B	3	5	H0YG33_HUMAN
ACSL3	Long-chain-fatty-acid--CoA ligase 3	2	7	ACSL3_HUMAN
HSPA5*	78 kDa glucose-regulated protein	6	4	GRP78_HUMAN
ALB*	Serum albumin	0	10	E7ESU5_HUMAN
ATP5A1*	ATP synthase subunit alpha	2	4	A8K092_HUMAN
TUBB4B*	Tubulin beta-4B chain	4	7	TBB4B_HUMAN
SLC25A6*	ADP/ATP translocase 3	3	4	ADT3_HUMAN
STOM	Erythrocyte band 7 integral membrane protein	4	3	B4E2V5_HUMAN
REEP5	Receptor expression-enhancing protein 5	2	4	REEP5_HUMAN
HSD17B12	Estradiol 17-beta-dehydrogenase 12	2	3	DHB12_HUMAN
TMEM33	Transmembrane protein 33	2	5	TMM33_HUMAN
TFRC	Transferrin receptor protein 1	4	2	TFR1_HUMAN
SLC7A5	Large neutral amino acids transporter small subunit 1	2	1	LAT1_HUMAN
LRRC59	Leucine-rich repeat-containing protein 59	2	1	LRC59_HUMAN
SCCPDH	Saccharopine dehydrogenase-like oxidoreductase	1	1	LRC59_HUMAN

* Likely contaminating protein typical of affinity purified LC-MS/MS (based on the CRAPome database). Highlighting: Grey = LAPTM4b bait protein. Yellow = 4F2hc and LAT1

Mass spectrometry (MS) identification of LAPTM4b associated proteins. Top hits of LAPTM4b binding partners identified by MS, with spectral counts indicated for two runs. Yellow highlights are 4F2hc (*SLC3A2*) and LAT1 (*SLC7A5*). LAPTM4b is highlighted in grey. Asterisks represent commonly identified MS contaminants (listed in the CRAPome database, http://www.crapome.org/).

Supplementary Table 2: Primers used in the study

Construct	Oligonucleotide
V2LHS_175**452**	TTGCAACCAACATGTTCAG
V3LHS_340**114**	TTGCTGGTAACATAAACCA
V3LHS_405**603**	AGCATTTCAGCAACAAACA
LAPTM4b pPCR Primer	F1 = 5’-TGTTACCAGCAATGACACTACG-3’<br>R1 = 5’-ATGTCTGCAAAGTCAAGCTG-3’
ΔC-LAPTM4b	F- 5’-AAAAAGCAGGCTTCATGAAGATGGTCGCGCCCTGG<br>R- 5’-AGAAAGCTGGGTTTATCGGTAGCAGTTCCAAACA
ΔN-LAPTM4b	F= 5’-AAAAAGCAGGCTTCGTCCGCACCGGCACCATC<br>R= 5’-AAGAAAGCTGGGTTTAGGCAGACACGTAAGGTGG

F=forward. R=reverse
